# Regulatory Elements and Splicing Factors Govern Mutually Exclusive Splicing of *DmRdl* Exon 3

**DOI:** 10.3390/ijms27219493

**Published:** 2026-10-24

**Authors:** Zijiao Song, Yichi Zhang, Kaixiang Lin, Xiugen Wang, Yanan Duan, Jian Bao, Chris Bass, Chunqing Zhao

**Affiliations:** 1 State Key Laboratory of Agricultural and Forestry Biosecurity, College of Plant Protection, Nanjing Agricultural University, Nanjing 210095, China2023202068@stu.njau.edu.cn (Y.Z.);; 2 Centre for Ecology and Conservation, University of Exeter, Penryn Campus, Penryn, Cornwall TR10 9FE, UK

**Keywords:** exon 3, *DmRdl*, mutually exclusive alternative splicing, CG30122, Rbp1-like

## Abstract

Ionotropic γ-aminobutyric acid (GABA) receptors are major inhibitory receptors in insect central nervous systems, with their diversity partly generated by alternative splicing. In *Drosophila melanogaster*, mutually exclusive alternative splicing (MEAS) of the resistant to dieldrin gene (*Rdl*) exon 3 (E3) produces *Rdl-3a* and *Rdl-3b*, but its regulatory mechanism remains unclear. In the present study, two intronic regulatory elements (IREs) and one exonic regulatory element (ERE) were identified in the exon 2 (E2)–exon 4 (E4) region of *DmRdl* precursor mRNA (pre-mRNA). The IRE1 mutation decreased exon 3b (E3b) inclusion from 54.12% to 3.95%, whereas the IRE2 mutation increased E3b inclusion from 61.70% to 97.82%. The ERE mutation significantly increased E3b inclusion both *in vitro* and *in vivo*, while the IRE mutations were lethal *in vivo*. The results of RNA pull-down, mass spectrometry, and RNAi demonstrated that the splicing factors, CG30122 and Rbp1-like bind to the ERE and exert opposite effects on E3b inclusion. The results of STRING network analysis supported the functional interaction between CG30122 and Rbp1-like, while molecular docking provided structural support for their potential interaction with the ERE-containing RNA sequence. This study reveals the molecular regulatory network underlying the MEAS of *DmRdl* E3, and and provides a foundation for investigating other MEAS exons, such as E6 and E9 in insect *Rdl*.

## 1. Introduction

Mutually exclusive alternative splicing (MEAS) is a form of alternative splicing in which only one exon from a cluster of tandemly arranged alternative exons is retained in the mature mRNA [1]. It enables a single gene to encode multiple protein isoforms, expanding the diversity of the transcriptome and proteome, and is critical for cell differentiation, organismal development, and the stress response [2,3,4]. MEAS is common in gene families such as ion channels, cell adhesion molecules, and transcription factors, and is essential for their functional specificity [5]. For example, the *Drosophila melanogaster* down syndrome cell adhesion molecule gene (*Dscam*) contains 95 variable exons, and MEAS of exons 4, 6, 9, and 17 can potentially generate 38,016 distinct mRNA isoforms with potential impacts on neural wiring and immune defense [6].

The resistant to dieldrin gene (*Rdl*) encodes a subunit of the ionotropic γ-aminobutyric acid (GABA) receptor, which is a major inhibitory neurotransmitter receptor in the insect central nervous system (CNS) [7]. The GABA receptor precisely regulates neuronal signaling and is essential for maintaining the normal excitation–inhibition balance in the insect CNS [8]. Rdl is also the primary target site for several major classes of neurotoxic insecticides including cyclodienes (dieldrin) and phenylpyrazoles (fipronil), and mutation of Rdl subunit in insects can lead to of insects against insecticides [9,10].

Exon 3 (E3) of the Rdl subunit undergoes conserved MEAS and may impact insect physiology [11,12]. The MEAS of *Rdl* E3 was first reported in *D. melanogaster* and resulted in changes in residues upstream of loop D, which is proposed to form a connection between the ligand-binding and transmembrane domains [13]. MEAS of *Rdl* generates two splice variants, *Rdl-3a* and *Rdl-3b* [12,13,14,15], which increases the structural diversity of insect GABA receptors, and potentially contributes to insect nervous system function, growth and development [8,13,14,16,17]. Therefore, understanding the regulatory mechanism underlying MEAS of *DmRdl* E3 may provide insight into the molecular basis of functional diversity within the insect CNS.

To date, the steric hindrance and competing RNA secondary structure models have been recognized as mechanisms specialized for regulating MEAS, whereas RNA–protein interactions represent a more general mechanism that regulates alternative splicing [18,19,20]. Under the RNA–protein interaction mechanism, precise regulation of MEAS depends on the coordinated interplay between regulatory elements, including exonic and intronic splicing enhancers and silencers [21], and splicing factors such as heterogeneous nuclear ribonucleoproteins (hnRNPs) [22] and serine/arginine-rich (SR) proteins [23,24]. In general, splicing factors bind to regulatory elements to enhance or inhibit spliceosome recognition of splice sites, thereby controlling the initiation and selection of MEAS events.

In the present study, we systematically investigated the mechanism underlying MEAS of *DmRdl* E3 due to the role of Rdl in insecticide resistance [15]. The potential contributions of steric hindrance and competing RNA secondary structure were first evaluated, followed by an investigation of the regulatory role of RNA–protein interactions. Thereafter, two intronic regulatory elements (IRE1 and IRE2) and one exonic regulatory element (ERE) were identified and were found to affect the inclusion of exon 3a (E3a)/exon 3b (E3b) *in vivo*. Subsequently, two splicing factors interacting with the ERE, CG30122 and Rbp1-like, were identified by RNA pull-down, mass spectrometry (MS) assays, and RNAi assays. Our findings elucidate the molecular mechanism governing the MEAS of *DmRdl* E3 and lay a foundation for further elucidating the regulatory networks of MEAS in *DmRdl* involved in insect neural development and physiological homeostasis.

## 2. Results

### 2.1. Subsection Identification of IREs in DmRdl Pre-mRNA

The predicted distance between the predicted branch site and the 5′ splice site of *DmRdl* E3b was 1755 nt, exceeding the 60 nt threshold. In addition, IE3 is a conserved intronic element predicted to be required for forming all three RNA secondary structures; however, its deletion did not affect E3b selection (Figure S9). These results suggest that neither steric hindrance nor competing RNA secondary structures are the primary mechanism underlying MEAS of *DmRdl* E3. We therefore investigated the interactions between splicing factors and regulatory elements.

First, the intron between E3a and E4 was divided into five segments (segments 1 to 5), and the corresponding deletion constructs (E3a-E4-Intdel1 to E3a-E4-Intdel5) were individually generated (Figure S1A). Among these constructs, only E3a-E4-Intdel5, corresponding to deletion of segment 5, affected the MEAS of *DmRdl* E3 (Figure S1B). Segment 5 was then further divided into five subsegments (segments 51 to 55) (Figure S2), and subsegment 55 was further divided into two smaller segments (segments 551 and 552) (Figure S3A) to identify the core nucletotide sequence. The results showed that the deletion of segment 552 disrupted the MEAS of *DmRdl* E3 (Figure S3B), suggesting that the key regulatory element resides within this segment.

**Figure 1:**
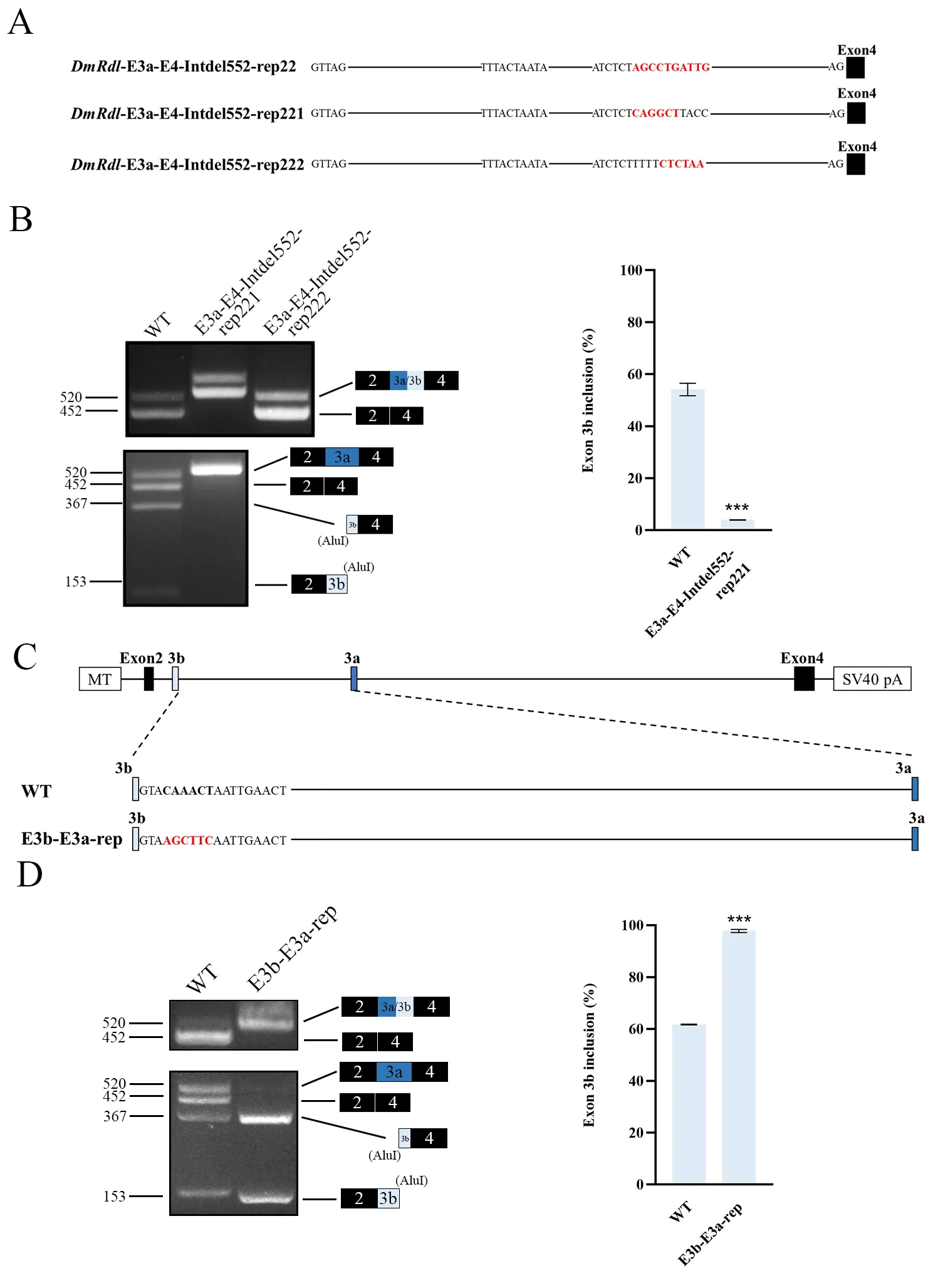
Identification of IREs mediating MEAS of *DmRdl* E3. Schematic of the replacement constructs E3a-E4-Intdel552-rep221 and E3a-E4-Intdel552-rep222 (**A**) and E3b-E3a-rep (**C**). Splicing patterns of the transfected E3a-E4-Intdel552-rep221 construct (**B**) and E3b-E3a-rep construct (**D**) in S2 cells, as detected by RT-PCR. The replaced region is indicated in red. The E3b inclusion level was calculated as the proportion of E3b relative to the total inclusion of E3b and E3a. Data are presented as mean ± standard error (SE) of three independent experiments. *** indicate *p* < 0.001.

Based on the sequence alignment of segment 552 from 16 *Drosophila* species, two conserved sequences, CS1 and CS2 (Figure S4A), were selected for further analysis to identify the core sequence for MEAS of *DmRdl* E3. CS2 was further subdivided and subjected to segment replacement (Figure S4B–E, Figure 1A,B), and a 6 nt sequence (Text S1) within CS2 was identified as the core sequence (Figure 1A), with replacement of IRE1 significantly decreasing E3b inclusion from 54.12% to 3.95% (Figure 1B).

Similarly, a regulatory element was identified in the intron between E3b and E3a of *DmRdl*. This intron was divided into seven segments (segment 1–7), and the corresponding deletion constructs (E3b-E3a-Intdel1 to E3b-E3a-Intdel7) were generated individually (Figure S5A). Deletion of segments 1 and 2 affected the MEAS of *DmRdl* E3 (Figure S5B). Based on the sequence alignment and the identification of overlapping regions among segments 1-3, a 6 nt sequence was identified and designated IRE2 as a potential regulatory element (Figure 1C), replacement of IRE2 significantly increased E3b inclusion from 61.70% to 97.82% (Figure 1D).

### 2.2. Identification of EREs in DmRdl Pre-mRNA

**Figure 2:**
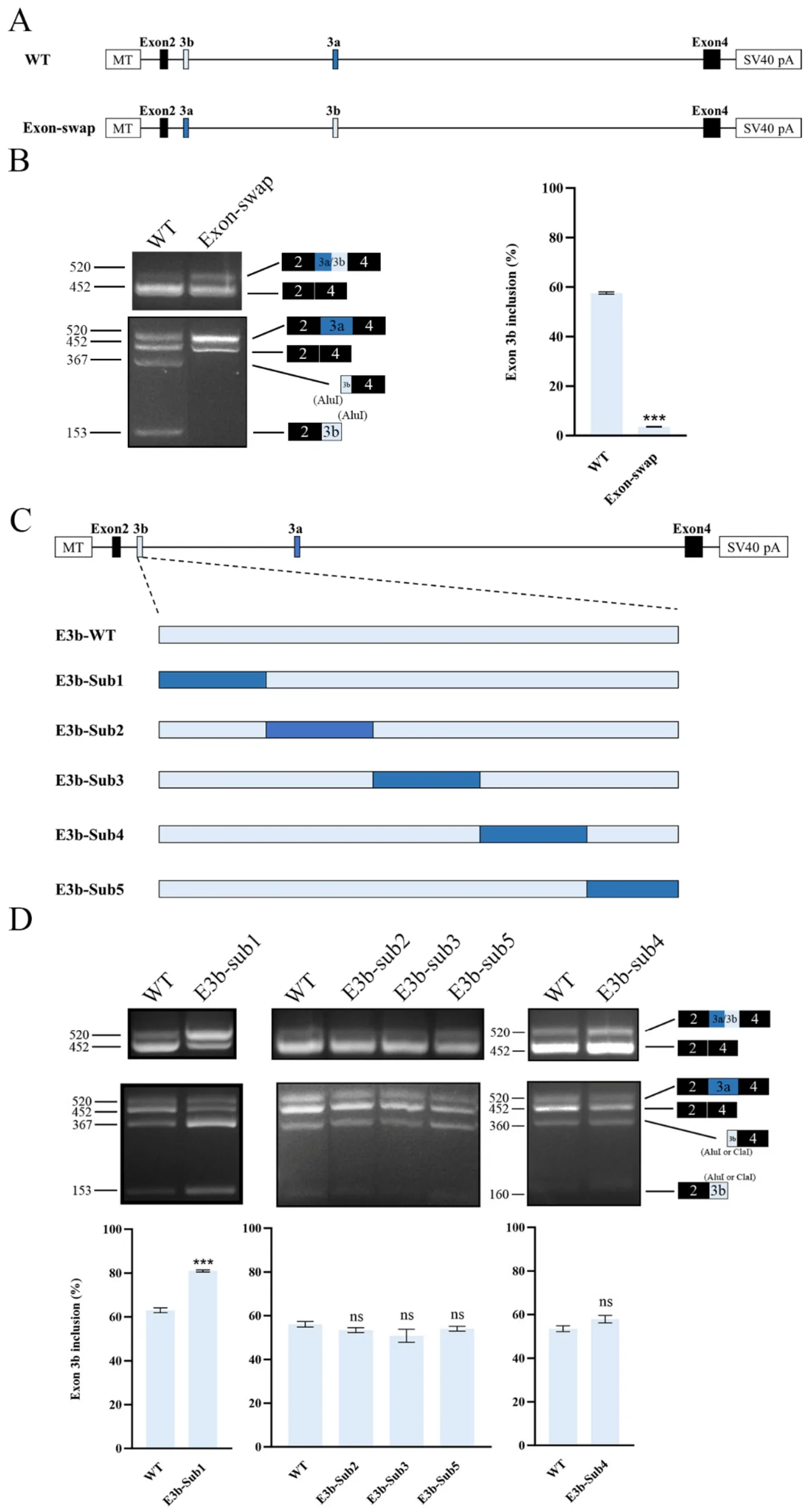
Identification of an ERE mediating MEAS of *DmRdl* E3. Schematic of the WT, Exon-swap constructs (**A**) and the substitution constructs generated from E3b (**C**). Splicing patterns of the transfected WT and Exon-swap constructs (**B**) and substitution constructs (**D**) in S2 cells, as detected by RT-PCR. Detailed information for the mutant series is shown in Figure S6. The exon inclusion level was calculated by comparing E3b or E3a to the sum of E3b and E3a. Data are presented as mean ± standard error (SE) of three independent experiments. ns indicates no significant difference (*p* > 0.05), *** indicate *p* < 0.001.

To identify EREs in E3, E3b and E3a were swapped (Figure 2A). As a result, E3b inclusion was almost abolished, decreasing from 57.57% to 3.56% (Figure 2B), suggesting that an ERE resides within E3b or E3a and contributes to the MEAS of *DmRdl* E3.

Subsequently, E3b was divided into five consecutive segments, each of which was replaced individually with the corresponding sequence from E3a (Figure 2C). Among these constructs, replacement of E3b-Sub1 (Figure S6) significantly increased E3b inclusion from 63.02% to 81.00% (Figure 2D), and E3b-Sub1 region was therefore defined as an ERE.

### 2.3. In Vivo Validation of the Regulatory Elements in MEAS of DmRdl E3

**Figure 3:**
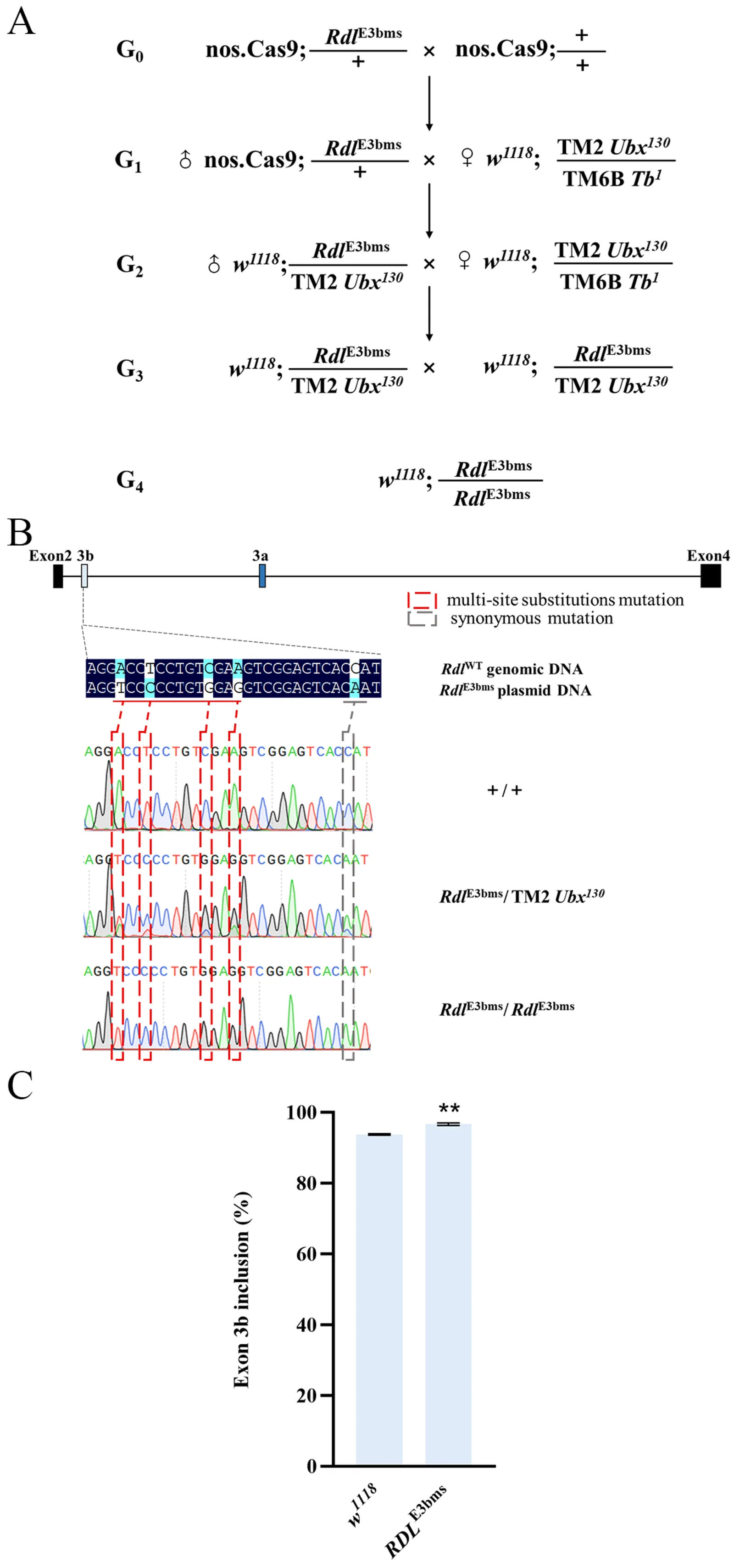
Generation and validation of *Rdl*^E3bm^*^s^* by CRISPR/Cas9 genome editing. (**A**) Crossing procedure used to establish homozygous *Rdl*^E3bm^*^s^* flies. (**B**) Genomic DNA sequencing of heterozygous and homozygous *Rdl*^E3bm^*^s^* flies. (**C**) Inclusion levels of *DmRdl* E3b and E3a in *w^1118^* and *Rdl*^E3bm^*^s^* flies. ** indicate *p* < 0.01.

To investigate the *in vivo* function of IRE1, IRE2, and ERE, we generated the *Rdl*^E3a-E4-rep^, *Rdl*^E3b-E3a-rep^, and *Rdl*^E3bms^ fly strains via CRISPR/Cas9. For the *Rdl*^E3bms^ fly strain, 800 G_0_ embryos were injected and 32 adults emerged. These 32 G_0_ adults were then individually crossed with nos.Cas9 flies. After G_1_ eclosion, individuals carrying the *Rdl*^E3bms^ allele were identified by sequencing. Among the 23 G_0_ crossed vials that produced G_1_ progeny, only one cross vial was positive for the *Rdl*^E3bms^ allele. The remaining 30 G_1_ male flies from this positive line were individually crossed to carrying the balancer chromosome to screen for positive heterozygous individuals (Figure 3A). Among the G_1_ parents that successfully produced G_2_ progeny, four carried the *Rdl*^E3bms^ allele. Positive G_2_ flies carrying the *Rdl*^E3bms^ allele in trans with the TM2 *Ubx*^130^/TM6B *Tb*^1^ balancer were intercrossed to obtain homozygous mutant G_3_ progeny. Homozygous mutant flies were then isolated from the G_3_ offspring, thereby establishing the homozygous *Rdl*^E3bms^ line (Figure 3B).

The same procedures were used to establish the *Rdl*^E3a-E4-rep^, *Rdl*^E3b-E3a-rep^ fly strains (Table S1, Figures S7 and S8). Unexpectedly, homozygous lethality was observed in both lines; therefore, RT-qPCR analysis was not performed for these mutants. The overall MEAS pattern in the homozygous *Rdl*^E3bms^ line was consistent with that observed in S2 cells, while E3b inclusion was significantly increased (Figure 3C).

### 2.4. RNA Pull-Down to Screen Splicing Factors Bound to the ERE

**Figure 4:**
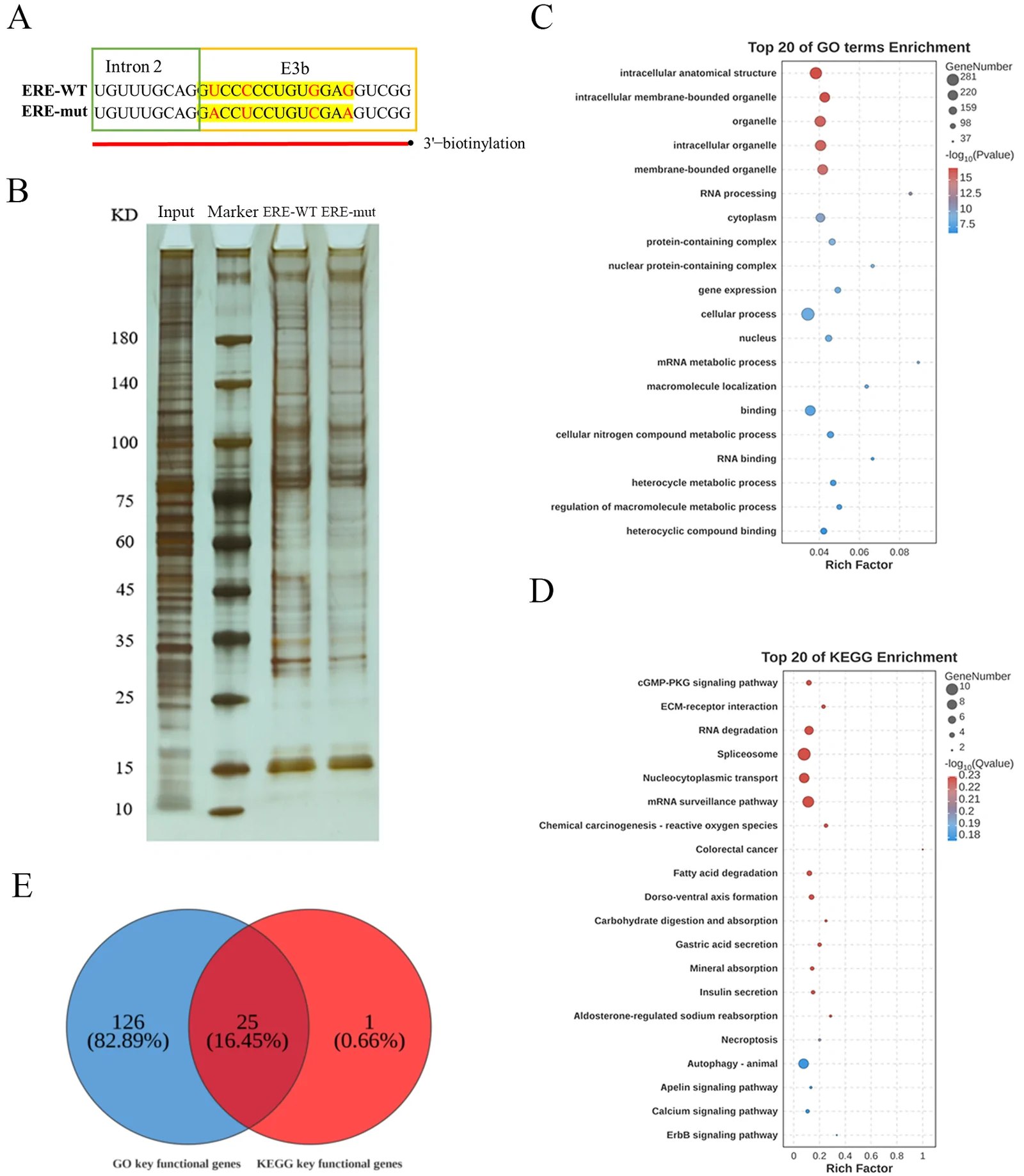
Identification of potential ERE-binding splicing factors involved in the MEAS of *DmRdl* E3. (**A**) Sequences of the ERE-WT and ERE-mut RNA probes. The ERE is highlighted in yellow, whereas sequence differences between the two probes are shown in red. (**B**) Proteins isolated by RNA pull-down were separated by 10% SDS-PAGE and visualized by silver staining. (**C**) GO and (**D**) KEGG enrichment analysis of DEP-associated genes identified by mass spectrometry after RNA pull down. Dot size represents the number of enriched genes, color intensity represents enrichment significance (−log_10_(*p*-value)), and the *x*-axis indicates the rich factor. (**E**) Venn diagram showing the overlap between genes enriched in key GO terms and KEGG pathways.

The *Rdl*^E3a-E4-rep^ and *Rdl*^E3b-E3a-rep^ strains were homozygous lethal, therefore, the subsequent analyses focused exclusively on the ERE. Both *in vitro* and *in vivo* experiments confirmed that the ERE plays a critical role in MEAS of *DmRdl* E3. To identify potential splicing factors involved in MEAS by binding to ERE, RNA pull-down assays were performed using ERE-WT and ERE-mut probes (Figure 4A). The results showed that the ERE-WT probe captured more of the cellular proteins than the ERE-mut probe (Figure 4B). Mass spectrometry analysis identified a total of 343 differentially enriched proteins (DEPs) that differed significantly between the ERE-WT and ERE-mut pull-down conditions (FDR ≤ 0.05 and |log_2_(FC| ≥ 1; Table S2).

GO enrichment analysis showed that the DEP-associated genes were mainly enriched in GO terms related to intracellular anatomical structures and organelles, including intracellular anatomical structure, intracellular membrane-bounded organelle and organelle, as well as in four RNA- associated GO terms, such as RNA processing, nuclear protein-containing complex, mRNA metabolic process, and RNA binding (Figure 4C). To further narrow down the candidate splicing factors, intersection analysis of these four RNA-associated GO terms identified 141 overlapping genes for subsequent functional validation.

KEGG enrichment analysis showed that the DEP-associated genes were mostly enriched in pathways related to RNA metabolism and splicing (such as RNA degradation, spliceosome, and mRNA surveillance pathways), as well as in other pathways, such as the cGMP-PKG signaling pathway (Figure 4D). To further narrow down candidate splicing factors, 26 overlapping genes for subsequent functional validation were identified by intersection analysis of three RNA metabolism and splicing pathways.

In the Venn diagram analysis of genes enriched in key GO functional terms and KEGG pathways, 25 overlapping genes were identified between them (Figure 4E) and four potential ERE-binding splicing factors, CG30122, Hrb97D, Rb97D, and Rbp1-like, were selected for subsequent functional validation based on their annotation as members of the hnRNP or SR protein families, two canonical classes of splicing factors.

### 2.5. CG30122 and Rbp1-like Bind to ERE to Regulate the MEAS of DmRdl E3

**Figure 5:**
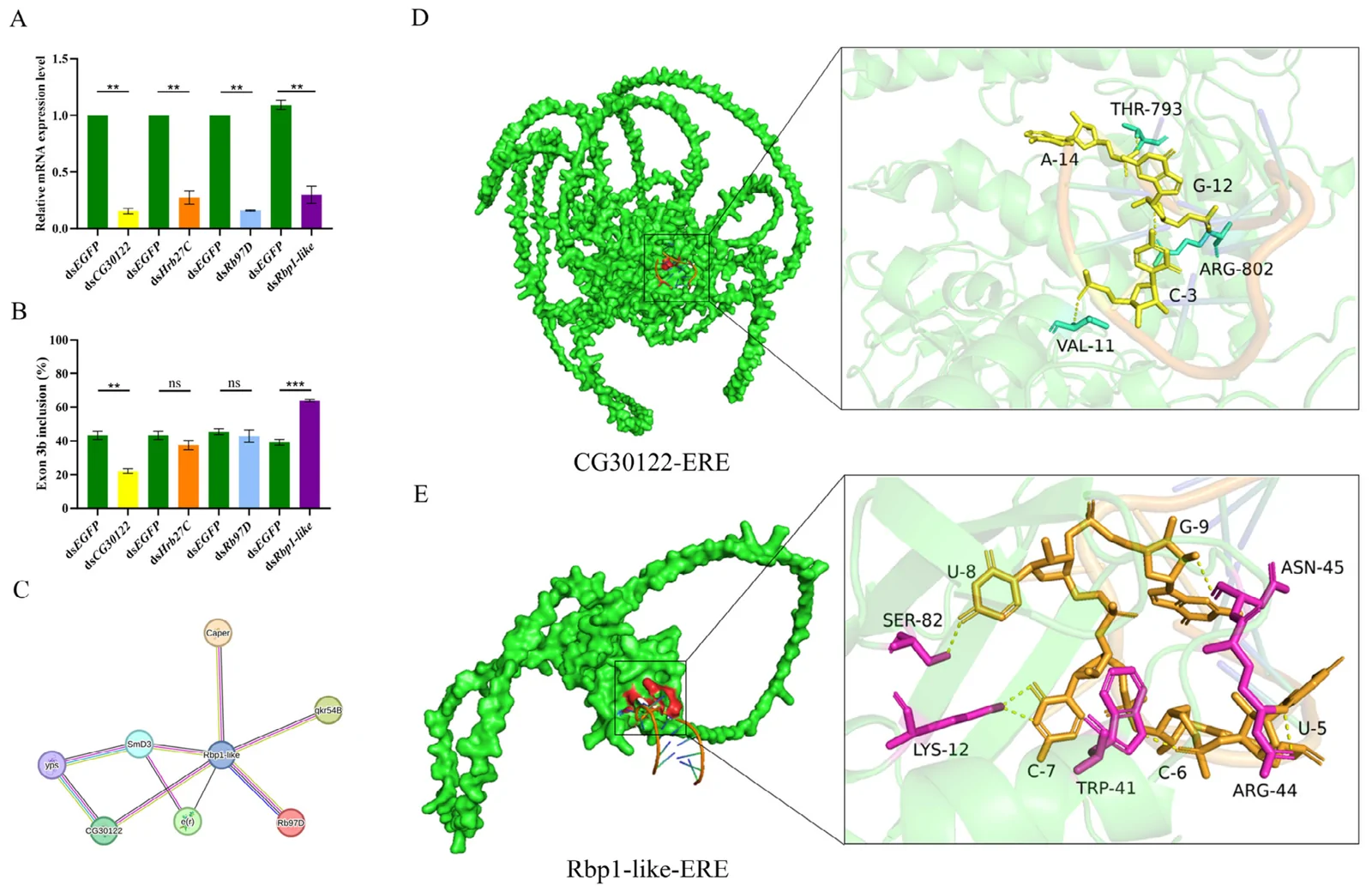
Identification and functional characterization of the splicing factors regulating *DmRdl* E3. (**A**) Relative mRNA expression levels of splicing factors following RNAi. The ds*EGFP* treatment served as a control. (**B**) Inclusion of *DmRdl* E3b 48h after RNAi. (**C**) PPI network generated using the STRING database. Molecular docking model showing the predicted binding of Rbp1-like protein (**D**) and CG30122 protein (**E**) to the *DmRdl* ERE. The yellow dashed lines represent hydrogen bond interactions. Error bars represent the mean ± standard error (SE) of three biological replicates. ns indicates no significant difference (*p* > 0.05). **, and *** indicate *p* < 0.01 and *p* < 0.001.

To investigate the regulatory role of four potential ERE-binding splicing factors (CG30122, Hrb97D, Rb97D, and Rbp1-like) *in vitro*, functional splicing assays were performed in S2 cells using RNAi. The mRNA levels of four potential splicing factor genes were significantly reduced after RNAi treatment (*p* < 0.01; Figure 5A). However, E3b inclusion in *DmRdl* transcripts was significantly affected only by knockdown of *CG30122* and *Rbp1-like*, and *CG30122.* E3b inclusion significantly decreased from 43.41% to 22.18% upon knockdown of *CG30122*, whereas it significantly increased E3b inclusion from 39.19% to 64.13% upon knockdown of *Rbp1-like* (Figure 5B), which indicated that CG30122 and Rbp1-like exert opposing effects on the MEAS of *DmRdl* E3.

To examine the functional relationship between CG30122 and Rbp1-like, STRING network analysis was performed (Figure 5C). The resulting network showed that CG30122 was associated with Rbp1-like and yps, and Rbp1-like occupied a central position and was connected to multiple RNA-processing and splicing-related proteins, including CG30122, SmD3, Rb97D, Caper and qkr54B. These results suggest that CG30122 and Rbp1-like canparticipate in the MEAS of *DmRdl* E3 through distinct but interconnected regulatory networks.

The ERRAT scores of interaction models, Rbp1-like–ERE and CG30122–ERE, were 90.698 and 85.235, respectively. In addition, 78.10% and 69.40% of residues in the two interaction models were located in the most favored regions of the Ramachandran plot (Table S3). In the predicted binding modes with ERE (Figure 5D,E), there were five hydrogen bonds identified at the Rbp1-like–ERE interface (ARG44: U5; TYR41: C6; LYS12: C7; SER82: U8; ASN45: G9), whereas three hydrogen bonds were identified at the CG30122–ERE interface (VAL11: C3; ARG802: G12; THR793: A14).

## 3. Discussion

**Figure 6:**
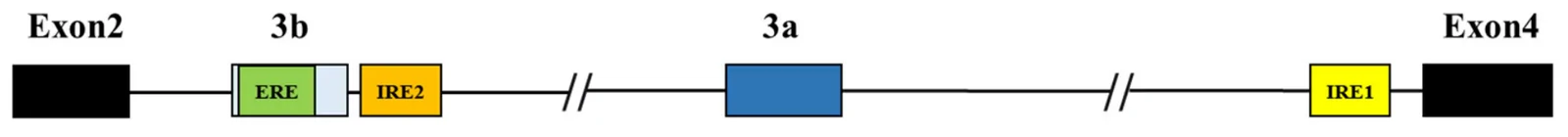
Schematic of regulatory elements (IRE1, IRE2, ERE) governing the MEAS of *DmRdl* E3.

Ionotropic GABA receptors are the major inhibitory neurotransmitter receptors in the insect CNS and important targets of insecticides. The MEAS of *DmRdl* E3 increases the diversity of the GABA receptor and thereby plays a role in regulating their physiological function [11,15]. In the previous studies, steric hindrance and competing RNA secondary structures are able to regulate MEAS in several genes [19,25,26,27,28,29,30]; our results do not support either mechanism as a primary determinant of the *DmRdl* E3 MEAS (Figure S9). Furthermore, among the regulatory elements located between E2 and E4, the three key elements (IRE1, IRE2 and ERE) predictably cannot form stable pairwise RNA secondary structures, indicating that a competing RNA secondary structure mechanism is also unlikely to account for the MEAS of *DmRdl* E3. Therefore, we investigated the regulatory mechanism underlying *DmRdl* E3 by identifying the regulatory elements involved in E3 selection using RNA-protein interaction assays. Through sequential deletion, fragment replacement and substitution mutagenesis, the key regulatory elements (IRE1, IRE2 and ERE) were mapped to the core motifs ranging from 6–13 nt (Figure 6).

Mutation of the 6 nt IRE1, which is located 80 nt upstream of E4, caused a near-complete shift toward E3a inclusion, confirming its central role in the MEAS of *DmRdl* E3 (Figure 1A). Other studies have demonstrated that IREs still influence the recognition of distal splice sites through RNA-protein complexes, even if they are located far from alternative exons [25,26]. A typical example is the Fox-1 and Fox-2 proteins, which bind to the specific UGCAUG motif to regulate alternative splicing. This motif is highly enriched in the 400 nt downstream of nervous system-specific exons [27], and this long-range regulatory mode is consistent with the proposed function of the IRE1. Based on this long-range regulatory model, IRE1 may promote E3a inclusion by recruiting specific splicing factors.

The substantial increase in E3b inclusion following IRE2 mutation suggests that this element plays a key role in maintaining the balance of E3 selection. A similar positional arrangement has been reported for an IRE in *FGFR2*, which is likewise downstream of the 5′ splice site of exon IIIb [28]. Such positional similarity of IRE2 to the 5′ splice site suggests that IRE2 is likely at a key regulatory site for splicing regulation. Previous studies showed that IREs adjacent to splice sites often act by recruiting splicing repressors such as hnRNP A1 and PTB, thereby interfering with U1 snRNP recognition or by destabilizing the exon definition complex and ultimately repressing exon inclusion [29,30]. Because alternative exons generally possess weak splice sites, the location of IRE2 may allow it to occlude key recognition regions surrounding the 5′ splice site and inhibit its recognition. Upon mutation, the inhibitory effect is relieved, U1 snRNP binding is enhanced, and E3b becomes nearly constitutively included, resembling the regulatory mode of the IRE in *SMN2* exon 7 [29].

Both *Rdl*^E3a-E4-rep^ and *Rdl*^E3b-E3a-rep^ fly strains were homozygous lethal. The lethality is highly unlikely to be driven by off-target effects, as heterozygous mutants were viable and fertile, and the intended replacements were verified by Sanger sequencing. Therefore, we speculated that the homozygous lethality probably is due to the essential role of IRE1 and IRE2 in maintaining the correct E3a/E3b balance. The IRE1 mutation nearly abolished E3b inclusion from 54.12% to 3.95%, whereas the IRE2 mutation increased it to near-constitutive inclusion (97.82%). Such extreme changes would drastically alter the ratio of E3a- and E3b-containing *Rdl* transcripts. Because different *Rdl* splice variants differ in GABA sensitivity [16], and E3 encoding residues contribute to the ligand-binding domain, this imbalance would impair receptor function and cause lethality. Consistent with this result, deletion of a GATA1-binding site in *ALAS2* intron 1 disrupts a long-range enhancer loop and causes embryonic lethality in mice [31]. Thus, the homozygous lethality of these mutants provides strong evidence *in vivo* that the precise regulation of *DmRdl* E3 splicing is essential for viability.

In this study, the identified ERE was located at the 5′ end of E3b and adjacent to the 3′ splice acceptor site, and exhibited a consistent splicing regulatory effect in both S2 cells and *D. melanogaster* *in vivo*. Mutation of the ERE significantly promoted E3b inclusion in both systems. E3b is already the predominant isoform in wild-type *D. melanogaster* (93.75 ± 0.24%), whereas E3a is expressed at a very lower level (6.25 ± 0.24%). Because these isoforms are mutually exclusive, the modest absolute increase in E3b inclusion is accompanied by an equivalent absolute decrease in E3a, representing a substantial relative reduction in the low-abundance E3a isoform. Thus, the ERE likely fine-tunes the E3a/E3b balance *in vivo* rather than acting as a binary switch, in cooperation with the IREs and associated RNA-binding proteins. Given its position, the ERE may influence 3’ splice site recognition, exon definition, and early spliceosome assembly. Short sequence variations near exon boundaries can markedly alter exon inclusion levels [32,33,34]. For instance, a single-nucleotide difference in *SMN2* exon 7 drastically shifts its splicing pattern toward exon 7 skipping, which constitutes a key molecular basis of spinal muscular atrophy [35]. This finding highlights that even extremely short exonic functional sequences can decisively affect alternative splicing. We speculated that, the position-mapping experiments, such as relocating the ERE within E3b, would further clarify the relative contributions of position and sequence to its regulatory activity.

Similarly, the subtle mutations near E10 of the *tau* markedly affect exon inclusion, further supporting the idea that sequences close to exon boundaries exert strong effects on exon selection [36]. Although EREs also reside in the central region of exons [35,37], those near the 3’ or 5’ splice sites are likely to exert stronger effects on exon inclusion. It is likely achieved through affecting splicing factors recruitment and local RNA–protein complex assembly. Although the ERE is only 13 nt in length, its location at the 5’ end of E3b likely confers strong splicing regulatory activity.

The RNA pull-down and RNAi results reveal that ERE can directly interact with CG30122 or Rbp1-like, which exert opposite effects on E3b inclusion. Specifically, E3b inclusion was significantly reduced by 21.23% upon knockdown of *CG30122*, whereas it was significantly increased by 24.94% upon knockdown of *Rbp1-like*. This suggests that the ERE is not merely a simple binding site mediating unidirectional control, but also a key regulatory element that integrates the opposite effects of two RNA-binding proteins. In wild-type *D*. *melanogaster*, E3b predominates over E3a. In S2 cells, the basal level of E3b inclusion is lower (43.41 ± 2.49%), but E3b inclusion was reduced by 21.23% upon knockdown of CG30122, whereas it was increased by 24.94% upon knockdown of *Rbp1-like*, substantially shifting the E3a/E3b balance. *Rdl*-3a and *Rdl*-3b are functionally different. For example, *LsRdl*-3a6b is more sensitive to fipronil than *LsRdl*-3c6b in *Laodelphax striatellus*, and *DmRdl*-3b6b has a higher GABA EC_50_ than *DmRdl*-3a6b in *D. melanogaster*. Thus, the modest changes in E3b may affect receptor function and insecticide sensitivity, with physiological and toxicological relevance. Similar regulatory principles have also been reported in other alternative splicing systems [38]. For example, in *SMN2* exon 7, the exon inclusion is determined by the opposing activities of SRSF1 and hnRNP A1, which promotes and repress inclusion, respectively. Ultimately, the final splicing outcome depends on the relative occupancy and activities of SRSF1 and hnRNP A1on the regulatory region [35].

Although the RNAi-induced change in E3b inclusion was moderate (~20%), it was statistically significant, and the two factors exerted opposite effects, consistent with their opposing roles in E3 selection. In S2 cells, E3b inclusion was approximately 40–60% after transfection with the WT plasmid; therefore, an absolute change of ~20% represents a substantial shift in the E3a/E3b balance. Regarding potential off-target effects, the dsRNAs were designed to be gene-specific, and the two knockdowns produced opposite effects on E3b inclusion, which is difficult to explain by a common off-target mechanism. In addition, RNA pull-down independently confirmed that both CG30122 and Rbp1-like bind the ERE, providing direct biochemical evidence for their involvement. Although off-target effects cannot be entirely excluded, the functional and biochemical evidence together supports the specificity of the observed splicing changes.

Likewise, in the mutually exclusive selection of *FGFR2* exons IIIb and IIIc, the same local pre-mRNA regulatory region can be modulated by Fox family proteins, hnRNP proteins, and other auxiliary factors, to generate distinct exon selection outcomes in different cell types [39]. Similarly, the classical splicing regulation of *Drosophila doublesex* also depends on the cooperative assembly of multiple RNA-binding proteins at EREs [40]. In our study, STRING interaction network analysis showed that Rbp1-like is linked to CG30122, as well as to spliceosome-related proteins such as SmD3 (Figure 5C), suggesting that these factors may participate in a dynamic protein complex that competitively or cooperatively associates with the ERE, thereby influencing early spliceosome assembly and exon recognition.

Molecular docking results suggested that Rbp1-like and CG30122 bind spatially adjacent but not fully overlapping sites on the ERE (Figure 5D,E) and Rbp1-like mainly occupied the middle region, whereas CG30122 preferentially bound for both ends. This spatial arrangement may affect local ERE conformation and the binding of other RNA-binding proteins. Thus, this ERE is a composite regulatory element that integrates the opposing effects of multiple RNA-binding proteins.

In summary, this study explored and excluded roles for the steric hindrance and competing RNA secondary structure models in the MEAS of *DmRdl* E3, systematically identified two IREs and one ERE in the *DmRdl* pre-mRNA, and verified the function of the ERE *in vivo* using the CRISPR/Cas9 system. Furthermore, RNA pull-down combined with RNAi confirmed that CG30122 and Rbp1-like bind to this ERE, and thereby regulate the MEAS of *DmRdl* E3. Collectively these findings provide new insights into the mechanisms underpinning transcriptomic diversity in an important neuronal receptor involved in diverse processes and a key target of insect pest control agents.

## 4. Materials and Methods

### 4.1. S2 Cell Culture and Drosophila Strains

*Drosophila* S2 cells were cultured at 27 °C in Schneider’s *Drosophila* Medium (Thermo Fisher Scientific Inc., Waltham, MA, USA) supplemented with 10% heat-inactivated fetal bovine serum (FBS, Thermo Fisher Scientific Inc.) and 1% penicillin–streptomycin (Thermo Fisher Scientific Inc.).

The *Drosophila* strains *w^1118^*, nos.Cas9 (stock #54591; Bloomington *Drosophila* Stock Center), and TM2 *Ubx*^130^/TM6B *Tb*^1^ (stock #FWB00002; Fungene Biotech, Qidong, China) were used as the wild-type (WT) strain, the zeroth generation (G_0_) strain for genome editing, and the balancer strain, respectively. All *Drosophila* strains were maintained on a standard fly diet at 25 °C under a relative humidity of 60–70%, and a photoperiod of 12:12 h (L/D) [41].

### 4.2. Prediction of Splice Branch Site, and RNA Secondary Structures

In tests of the steric hindrance model, the splice branch site for intron between E3b and E3a in the *DmRdl* pre-mRNA (Figure S10) was predicted using the LaBranchoR tool (https://github.com/jpaggi/LaBranchoR, accessed on 18 October 2024) with a 70 nt sequence upstream of the 3′ splice site as input, as described in a previous report [42]. If the distance between the 5’ splice site and the splice branch site exceeded 60 nt, the steric hindrance model was considered unlikely to account for MEAS.

In tests of the competing RNA secondary structure model, genomic *Rdl* sequences from 16 *Drosophila* species belonging to the family Drosophilidae were retrieved from the National Center for Biotechnology Information (NCBI) database (Table S4) and aligned using the MEGA-X software (v10.2.0) [43] to identify the conserved sequences, which were then used to predict RNA secondary structures with RNA Folding Form V2.3 (https://www.unafold.org/mfold/applications/rna-folding-form-v2.php, accessed on 30 October 2024) [44].

### 4.3. Plasmid Construction and Transfection of S2 Cells

A genomic fragment (Figure S10) of the *DmRdl*, including E2, exon 4 (E4), and the complete intronic and exonic (e.g., E3a and E3b) sequences between E2 and E4, including E3a and E3b, was amplified by PCR from the genomic DNA of *w*^1118^, and used as the wild-type (WT) minigene. Subsequently, the WT minigene was inserted into the pMT/V5-HisB vector (Thermo Fisher Scientific Inc.) via the KpnI and ApaI sites using the ClonExpress II One Step Cloning Kit (Vazyme Biotech Co., Ltd., Nanjing, China) to generate the WT construct.

To disrupt RNA secondary structures and precisely identify the regulatory element positions, we adopted a strategy combining stepwise segmental deletion and fragment replacement of conserved regions. Then, a series of deletion or replacement constructs (Text S1 and Table S5) were introduced into the WT construct using PCR-based site-directed mutagenesis with specific primers (Table S6). All constructs were verified by Sanger sequencing at General Biol Anhui Co., Ltd., Chuzhou, China.

To generate the Exon-swap construct, the E3b in the WT construct was exchanged with E3a using homologous arms and PCR (Table S6), followed by homologous recombination with the ClonExpress II One Step Cloning Kit. The same procedure was subsequently used to replace exon 3a with exon 3b. A series of substitution constructs (E3b-Sub1 to E3b-Sub5) were generated by swapping E3b with subregions of E3a using PCR-based site-directed mutagenesis (Table S6).

The WT, deletion, or substitution constructs (Table S5) were transfected into S2 cells using Lipofectamine 3000 Transfection Reagent (Thermo Fisher Scientific Inc.). After 5 h post-transfection, CuSO_4_ (100 mM) was added to activate the metallothionein promoter and thereby induce minigene expression. The cells were harvested 48 h after transfection.

### 4.4. Quantification of mRNA Splice Isoforms

Total RNA from S2 cells transfected with each construct was extracted using TRIzol Reagent (Thermo Fisher Scientific Inc.). First-strand cDNA was synthesized by reverse transcription using the PrimeScript II 1st Strand cDNA Synthesis Kit (Takara Bio. Inc., Kusatsu, Japan). The reaction system for RT-PCR contained 12.5 μL of 2× Phanta^®^ Master Mix, 1 μL of cDNA template, 1 μL of *DmRdl*-det-F and -R primers (10 µM), and 9 μL of ddH_2_O. The PCR procedure was as follows: 95 °C for 3 min; 34 cycles of 95 °C for 15 s, 61 °C for 15 s, 72 °C for 30 s; followed by a final elongation at 72 °C for 5 min. PCR products were digested with exon-specific restriction enzymes (AluI was used for all constructs except the E3b-Sub4 construct, for which ClaI was used) and detected by 3% agarose gel electrophoresis (Figure S11). Images were captured using the Tanon-6600 Gel Imaging System, and the mRNA isoforms were quantified by analyzing the gray values of the DNA bands using ImageJ software (v1.52a;Bethesda, MD, USA) [45]. Data were expressed as percentages of mean and SE, and the error bars were calculated from three biological replicates. The significance of differences was determined by the two-tailed Student’s *t*-test, and *p* < 0.05 was deemed to indicate statistical significance.

### 4.5. CRISPR Design and Plasmid Construction

Potential CRISPR target sequences within the genomic region were identified using the online platform CRISPR Optimal Target Finder (http://tools.flycrispr.molbio.wisc.edu/targetFinder/, accessed on 8 May 2025). Two sgRNA target sequences without predicted off-target sites (Table S7) were selected and cloned into the pCFD4 vector backbone (Addgene#49411) [46] using specific primers (Table S7) to generate the sgRNA plasmid.

Three ~2.5 kb PCR products containing IRE1, IRE2 or ERE and their respective ~1.2 kb upstream and downstream sequences, were amplified from the genomic DNA of the nos. Cas9 strain as the template using specific primers (Table S7). The resulting fragments were ligated into the *pEASY*-Blunt3 vector (TransGen Biotech, Beijing, China) to generate *pEASY*-Blunt3-IRE1, *pEASY*-Blunt3-IRE2, and *pEASY*-Blunt3-ERE, respectively.

In *pEASY*-Blunt3-IRE1, *pEASY*-Blunt3-IRE2, and *pEASY*-Blunt3-ERE, IRE1, IRE2, and ERE were replaced with the corresponding replacement fragments E3a-E4-Intdel552-rep221, E3b-E3a-rep and E3b-Sub1, respectively. In addition, synonymous mutations were introduced into the protospacer adjacent motif (PAM) sequences to improve the efficiency of editing [47] using the Fast Mutagenesis System (TransGen Biotech) with specific primers (Table S7), thereby generating the donor plasmid for embryo microinjection. The resulting genome-edited fly strains were designated *Rdl*^E3a-E4-rep^, *Rdl*^E3b-E3a-rep^, and *Rdl*^E3bms^, corresponding to replacement of IRE1, replacement of IRE2, and multiple substitutions in E3b within the ERE region, respectively.

### 4.6. Real-Time Quantitative Polymerase Chain Reaction (RT-qPCR)

For gene expression analysis, total RNA was extracted from S2 cells subjected to RNAi treatment with ds*CG30122*, ds*Hrb97D*, ds*Rb97D* and ds*Rbp1-like*, as well as from the *Rdl*^E3bms^ and *w*^1118^ strain, respectively and reverse-transcribed into cDNA. RT-qPCR was performed in a 20 μL reaction mixture containing 2 μL of cDNA template, 10 μL of 2× ChamQ Universal SYBR qPCR Master Mix (Vazyme Biotech Co., Ltd.), 0.4 μL each of forward and reverse primers (10 μM; Table S8), designed with Beacon Designer 7 (Premier Biosoft International, Palo Alto, CA, USA), and 7.2 μL of nuclease-free water. Amplification was performed on an ABI 7500 system under the following conditions: 95 °C for 30 s, 40 cycles of 95 °C for 5 s and 60 °C for 34 s, followed by a melting curve program of 95 °C for 15 s, 60 °C for 60 s and 95 °C for 1 s. Three technical replicates were performed for each biological sample. The relative expression levels of E3b and E3a were normalized to *Actin* as the internal reference gene.

Data from RT-qPCR assays were exported from the ABI 7500 SDS 1.4 software (Thermo Fisher Scientific Inc.). The comparative cycle threshold (Ct) values were analyzed using Sequence Detection Software 1.4 (Thermo Fisher Scientific Inc.) and Excel 2016 (Microsoft). For fly samples, RT-qPCR was performed using isoform-specific primers for E3b and E3a (Table S8), with Actin as the internal reference. E3b inclusion was calculated by qPCR as described previously [48]. The relative abundance of E3 isoform (E3b or E3a) was calculated as 2^−ΔCT^, where Δ*Ct* = *Ct*_isoform_ − *Ct*_Actin._ E3b inclusion was then calculated as the relative abundance of E3b divided by the sum of the relative abundances of E3a and E3b, multiplied by 100%. For comparisons between fly strains, the calculated E3b inclusion percentages were used directly. For RNAi experiments, relative mRNA expression levels of target genes were calculated using the 2^−ΔΔCT^ method [49]. Statistical comparisons were performed using one-way analysis of variance (ANOVA) followed by Tukey’s multiple comparisons in IBM SPSS Statistic 22.0 software (SPSS Inc.). Figures were prepared using GraphPad Prism 6.0 (GraphPad Software Inc.).

### 4.7. RNA Pull-Down and LC-MS/MS

Two 3′-biotin-labeled RNA probes, the wild-type ERE probe (ERE-WT) and the mutant ERE probe (ERE-mut), in which the ERE sequence was replaced with the corresponding sequence present in the E3b-Sub1 substitution construct, were synthesized (GenScript Inc., Piscataway, NJ, USA) to capture RNA-protein complexes from whole-cell extracts of S2 cells transfected with the WT construct for 48 h.

Whole-cell extracts from S2 cells were prepared as previously described [50]. Three independent biological replicates were performed for each probe. The biotin-labeled RNA probe (1 μg) was incubated with 50 μL of Mag-SA streptavidin magnetic beads (Bioeast Inc., Ltd., Hangzhou, China) at 4 °C for 1 h. Subsequently, 400 μg of whole-cell extracts were added and incubated at 4 °C for an additional 1 h. After incubation, the beads were collected by magnetic separation and washed three times with ice-cold PBS, with the supernatant removed after each wash. Finally, 40 μL of 2× SDS loading buffer was added, and the samples were boiled for 10 min. The resulting RNA-binding proteins were separated by SDS-PAGE and visualized by silver staining to identify differential bands between ERE-WT probe and ERE-mut probe pull-down samples.

In addition, the resulting RNA-bound proteins were separated by SDS-PAGE and stained with Coomassie brilliant blue. The stained bands were then excised, cut into small pieces, and destained with 50% acetonitrile (ACN) in 50 mM ammonium bicarbonate. The gel pieces were subsequently reduced with 10 mM DTT for 30 min, alkylated with 10 mM iodoacetamide (Sigma, St. Louis, MO, USA) at room temperature for 20 min, and digested with trypsin (Promega Corporation, Madison, WI, USA). The resulting peptides were extracted from the gel, collected, desalted, dried, and redissolved for LC-MS/MS analysis.

### 4.8. Bioinformatic Analysis of DEPs

The resulting peptides were analyzed by LC-MS/MS, and the resulting protein abundance data were further analyzed using Perseus software (v2.1.6.0) [51]. After data normalization and filtering of low-abundance proteins, differential proteins between the ERE WT and ERE mut probe groups were identified using two-tailed Student’s t-test. The raw *p*-values were corrected using the Benjamini and Hochberg method to control the false discovery rate (FDR), and proteins with FDR ≤ 0.05 and |log_2_(fold change, FC)| ≥ 1 were defined as DEPs.

Gene Ontology (GO) and Kyoto Encyclopedia of Genes and Genomes (KEGG) enrichment analyses were performed using the OmicShare tools (http://www.omicshare.com/tools, accessed on 21 November 2025). The genes corresponding to the DEPs identified by RNA pull-down coupled with LC-MS/MS were mapped to GO terms in the GO database (http://www.geneontology.org/, accessed on 21 November 2025) and to KEGG pathway terms in the KEGG database (https://www.genome.jp/kegg/, accessed on 21 November 2025). Enrichment of GO terms and KEGG pathways was assessed against the *D. melanogaster* background gene set using hypergeometric tests. The resulting *p*-values were adjusted via false discovery rate (FDR) correction, with FDR ≤ 0.05 as the significance threshold.

To construct the protein–protein interaction (PPI) network, all identified DEPs were submitted to STRING version 11.5 (https://string-db.org/, accessed on 30 December 2025). The subset of DEPs predicted to interact with the two identified splicing factors was extracted and used to construct the PPI network with a medium confidence score threshold of 0.400. The resulting network was visualized and exported directly from the STRING platform.

Molecular docking between the splicing factor (Text S2) and ERE sequence of *DmRdl* pre-mRNA (Text S1) was performed using AlphaFold 3.0 (https://www.alphafoldserver.com, accessed on 19 February 2026). An inter-protein Template Modeling score (ipTM) > 0.6 was considered indicative of a meaningful interaction. The predicted complexes were further analyzed structurally and visualized using PyMOL (v1.8) [52].

### 4.9. RNA Interference (RNAi) Assay

Double-stranded RNA (dsRNA) targeting splicing factor (*CG30122*, *Hrb97D*, *Rb97D*, or *Rbp1-like*) was synthesized using the T7 RNAi Transcription Kit (Vazyme Biotech Co., Ltd.) from DNA templates flanked by T7 promoter sequences and amplified with specific primers (Table S8). S2 cells were seeded into 6-well plates and cultured to 60–80% confluence. For co-transfection, 2 μg of WT plasmid and 2 μg dsRNA were added to each well, with ds*EGFP* as the negative control. Subsequently, the expression levels of *DmRdl* E3 splice variants after RNAi was analyzed by RT-qPCR with *Actin* as internal references.

Total RNA was extracted after 48 h post-transfection and specific primers (Table S8) for RT-qPCR were designed based on the E3 sequence of *DmRdl*. RNA purification, cDNA synthesis, RT-qPCR data analysis, statistical analysis was performed as described above. Amplification efficiency was evaluated, and product specificity was confirmed by melting curve analysis. Three biological replicates were analyzed, each with three technical replicates.

## 5. Conclusions

This study systematically identified two IREs and one ERE in the *DmRdl* pre-mRNA and verified the function of the ERE *in vivo* using the CRISPR/Cas9 system. Furthermore, RNA pull-down combined with RNAi confirmed that CG30122 and Rbp1-like bind to this ERE and thereby regulate the MEAS of *DmRdl* E3. Collectively, these findings provide new insights into the mechanisms underpinning transcriptomic diversity in an important neuronal receptor involved in diverse processes and a key target of insect pest control agents.

## Data Availability

The raw data supporting the conclusions of this article will be made available by the authors on request, as the data are part of an ongoing study.

## Supplementary Materials

The following supporting information can be downloaded at: https://www.mdpi.com/article/10.3390/ijms27219493/s1.

## Abbreviations

The following abbreviations are used in this manuscript:

cDNA	complementary DNA
CNS	central nervous system
DEPs	differentially enriched proteins
ds*EGFP*	double-stranded RNA of enhanced green fluorescent protein
dsRNA	double-stranded RNA
E2	exon 2
E3	exon 3
E3b	exon 3b
E3a	exon 3a
E4	exon 4
FC	fold change
GABA	γ-aminobutyric acid
MEAS	mutually exclusive alternative splicing
pre-mRNA	precursor mRNA
qRT-PCR	quantitative reverse-transcription polymerase chain reaction
*Rdl*	resistance to dieldrin gene
Rdl	resistance to dieldrin protein
RNAi	RNA interference
SE	standard error
sgRNA	single-guide RNA
