# Supplementary material for: Regulatory Elements and Splicing Factors Govern Mutually Exclusive Splicing of DmRdl Exon 3
Source: Int J Mol Sci. 2026 Oct 24;27(21):9493. doi: 10.3390/ijms27219493 (PMC13762713; doi:10.3390/ijms27219493)
Supplement: Supplementary file 1 [file ijms-27-09493-s001.zip › Supplementary Information-20260726-R.pdf]

Supplementary Information

Title: Regulatory elements and splicing factors govern mutually exclusive splicing of *DmRdl* exon 3

The full names of all authors:

Zijiao Song [a#], Yichi Zhang [a#], Kaixiang Lin [a], Xiugen Wang [a], Yanan Duan [a], Jian Bao [a], Chris Bass [b], Chunqing Zhao [a,*]

The authors' institutional affiliations at which the work was carried out:

[a]. State Key Laboratory of Agricultural and Forestry Biosecurity, College of Plant Protection, Nanjing Agricultural University, Nanjing, 211800, P.R. China;

[b]. Centre for Ecology and Conservation, University of Exeter, Penryn Campus, Penryn, Cornwall TR10 9FE, United Kingdom;

****Corresponding author:*** Chunqing Zhao;

Tel: +86-176-0155-3101;

E-mail: zcq@njau.edu.cn;

Supporting Text

Text S1 Sequence annotation of WT construct

Constitutive exon sequences are highlighted in gray: the upstream sequence is E2 and the downstream sequence is E4. The regions upstream of E2 and downstream of E4 correspond to sequences from the pMT/V5-HisB vector. Alternative exon sequences are highlighted in blue: E3b in light blue and exon 3a in dark blue. Regulatory element IRE1, IRE2, and ERE are labeled in yellow, blue, and red font, respectively. Conserved intronic elements IE1, IE2, IE3, and IE4 are underlined in sequence order.

TCGCGCGTTTCGGTGATGACGGTGAAAACCTCTGACACATGCAGCTCCCGGA
GACGGTCACAGCTTGTCTGTAAGCGGATGCCGGGAGCAGACAAGCCCGTCA
GGGCGCGTCAGCGGGTGTTGGCGGGTGTCGGGGCTGGCTTAACTATGCGGCA
TCAGAGCAGATTGTACTGAGAGTGCACCATATGCGGTGTGAAATACCGCACA
GATGCGTAAGGAGAAAATACCGCATCAGGCGCCATTCGCCATTCAGGCTGC
GCAACTGTTGGGAAGGGCGATCGGTGCGGGCCTCTTCGCTATTACGCCAGCT
GGCGAAAGGGGGATGTGCTGCAAGGCGATTAAGTTGGGTAACGCCAGGGTT
TTCCCAGTCACGACGTTGTAAAACGACGGCCAGTGAATTAATTCGTTGCAGG
ACAGGATGTGGTGCCCGATGTGACTAGCTCTTTGCTGCAGGCCGTCCTATCCT
CTGGTTCCGATAAGAGACCCAGAACTCCGGCCCCCCACCGCCCACCGCCACC
CCCATACATATGTGGTACGCAAGTAAGAGTGCCTGCGCATGCCCCATGTGCC
CCACCAAGAGCTTTGCATCCCATACAAGTCCCCAAAGTGGAGAACCGAACC
AATTCTTCGCGGGCAGAACAAAAGCTTCTGCACACGTCTCCACTCGAATTTG
GAGCCGGCCGGCGTGTGCAAAAGAGGTGAATCGAACGAAAGACCCGTGTGT
AAAGCCGCGTTTCCAAAATGTATAAAACCGAGAGCATCTGGCCAATGTGCAT
CAGTTGTGGTCAGCAGCAAAATCAAGTGAATCATCTCAGTGCAACTAAAGGG
GGGATCTAGATCGGGGTACCGACTGGCGGTGGCAGCATGCTGGGTGACGTA
AACATATCCGCTATTCTCGACTCCTTTAGTGTTAGTTACGACAAAAGAGTAAG
ACCCAATTACGGTGGTAAGTAGACCAATCTCCGGACACCCAATCTTAATAGTT
ACACTTACCCAGCGAAAGCTAAAGCAAAAGAGCAAACCCAAAACGCAACG
CGACTTGAACGAAACGCAAGTGGGGGCACAGCCCGGTAACCAAAATGTATC

CAGTATATATAGGGAGGTTGAGGCGCAGTGGGATCTGAGATGTGGGAACTGG
GATCTGGGATCCGAGAACTTTAACACTAGACTAATCCTTTAAACTTTAAACCG
TACCTAGCTATATAACACCTACTATAACATACGTGTTTGCAGGACCTCCTGTCG
AAGTCGGAGTCACCATGTATGTGCTATCGATCAGCTCGCTCTCAGAAGTGAAA
ATGGTACAAACTAATTGAACTTTGTACCTTTCTATTATCGTTAAGCGCTCTCTCG
CATTCTATCTCTATCTATCTACTCCTAAATCTATCTCTCTGTCTTTGGTCTTTGTT
GTTGGAGTTTTCTTTCGAATTTTAATTTCCTTTCGTACCACAAAACGCATTAGTT
TGTTGCTCTCTCACACTCACACACTCACACACACAAAGTCTTGATTTCCTAATT
TCCATGGTACTACCAACTACCAAATGTTTCAACTGTTTTGCTTTGTATGAACTC
TCTCTCCAAACACTATTTTATATATATATATATATATATAGATAGAACGATCTATTAT
TTCTTGACCGTTGACCAAAGTACACCAGTTGAAAGCGTTATTTGCGAATCTTAT
CCACACCCGTACCTATTGTGTTTCCTTCTATTTATATGGTATTATTTTCTCATCAG
TTACCCTTTTGGTACTAAATCTCTCAAAAACTCTAACTCTTTCGAATGTTTAGTT
CGTTTTATAGCCGCTGTGTTTTAGTGTTTTTCCCAACAATTTCGCATTCATTTCC
TTAAATGAATACTTTAGCCAAGGTCTTTGTTAGGAGCTGACGAGCCATACGAA
CCCAGAAGCCAGAATAATAGGGCCAAATGTTATAGTACTTGCCACGATATTTT
TCTCACATCGTCAACACATTGTAAACTAAATGTCAACTCTCAAATCCATCAAA
ACCAACGATTGGCAAAAAGCTTTTTATGTTCCTTAAGCTCTACAAAATGACTT
CCTCCTCTGCTCACCGTTACTTTTAACTCTGTGTGTGTTTGTGTATGTCTAATTG
GCCAAGCACATCAAAATTACCAAAAAAAAAAATAAAAACAAAAACAAAAA
TGAAAATGAAAAACCTCACACATACAAACAACAGCCACAGTTACAAAACAA
ATCAATCAACATTGTACCCCTTCCGGATCTCAAAACCCTCACATTCCAAGCAA
AACTCTTGCCTCTCACTTCATGCGTATTGTTTCGTCTTCTTCTTTTTTGTAGGCC
CACATAACTCATAACAAGCTTCAAGATAAATTTCATTACCCAACTGCTGCCAA
AAATCGCAATACCCAAAATCCAAAAAAAAAAAAATACAAATCTCTATCTAAA
TAAGCAATGCAAACGAAAATGGTGTGTGAATCCGCTATTCAAAACCAAATAA
TCCACTAGTTCAAATAAATATTTATTTACTAGGCGCCTTTGTTTTTGAAATACTT
TCTTTTAATTGAGCTACTGCAACTCTTATTCGTATAGCTGAAAATACATGCAAA
ATATACAACATTAATGTGAACCTCGAGTAGAGTCAACTGATTTGGTTTTCATTC

GGCGCGTGCAATCAACAGAAGCAAAAGCATCAAACACAAATTTAGTTAGTCA
CTACAAAAAAAAAAAAAAAAATATAGACAGCCAAAACAGTACAACAACAGC
AAAGCAATGCACATCGCTTGCCAATTTAAGTGTTAAACGTACCTTATATATTTA
ATTTTGCTAATGGAAATTAAACAGCACCCGACGAGCACAAGGAAAGGCGCCC
GAAAGTAGGCAGCGAAAATAAAATATATGTCAACGTACTGCGTCCATTGTTAC
TACTCGTACCGCTGTGTGTGTGTTTCAGTTCTCATATTTTGGGTTTTAGCCACAT
CTAACATGAACAACTACAATAATAATAATAACTACAACAACAACAACAACTA
CAACTACAGTATAACCATACCAAACGCAAAATGAAAATTCCATCAATCCGTGT
GTGTGTATATGTTGCAGGTCCCCCTGTGGAGGTTGGCGTCACAATGTATGTCCT
CAGTATCAGTTCGGTTTCGGAAGTTCTAATGGTACATACGTAAAAATATATGCC
AAAAATACAAATAAATAAAAAAAACAAAAATTATCCACAGATACAAAACAA
ATGTTCCTTTTTTTTTTTGGTAAAAGCAAACAAAAAAAAATTCATAAATCTACT
CGTATACTTCTTAATTTCTCTCAGTCTCTACCTCTAACCTGTAACTATCTCTCGC
TCTCTGTCTCTATAAATATCTCTGTCTCCTTTACTATATGTTTCTATCTCTTTCTCT
ATGTCTGTCTCTGTTGCTTAAAGCTCATTTTGTGTCTTAGCATTTGTTCTCCTTTT
CATTTCGCTAATTGGCAACAGACATACAAATTTATTACAATTACTTTGATTCAC
TTAAGATTGTGTTTGTTTTCGACCATTCAAAGCCAACTGCAAACCAATATCTAT
ATACATATACCAATATCCTATATATACAAAGTTCTGCCAAATTAAATGTTGTGTA
AAACGAATCGTTATTGCAATGCAATTGCCAACTATGAAATTCCAATGTTGCAT
ATTCGTTTTGTGTTTATTGTTTCAATGTTAGCCAATAAGGCCATGATGTTCGCAA
ATTGTTTGTTGTTCCGTTTCAAAAGCTTCTCTCGGCCAGAACAATGCAAAACC
GAAATATATACAATATATATACTTACATTGAAATTACCCCAAATTATACACATTT
TGAGCTAATTTTTGAGCCCAGACCAAGTGCTGCTCTCTCTTTCACCCACACTC
GCGCTCTATCTCTATCTAATATTCAACTACGAGTTTGACGCTTTGAAATGCCATT
TCACCATTTGTTCATCCTGAGTTTGTTGCCTTTTGCGTTTCTTTGTCTCCATCGC
ATCCATCCACTTCGGAATTTTATGGATAACATGGATAACTGCGATAAAGTCAAT
CAATCTAATTGCTGTATAAATCTTTATTTATCGCCCCATCGAGTACATTACGGCC
AAAGTTCATCCATTTCCCCAACCCACATACACAGTGACACTATGATGGGTTCC
CTCCACCCCAGAGAAACGAGAGATTTTTATGACATATTCATGCATACATATATC

CATCATACCTATACTCGCATATAGCCCTCATTATGTTTGTTGTGATCTGGTCAAA
TGCAAAATATCAAAATTAATTAAATAAAATTAATCTCAGCGGCCGCATTGACA
AAGGCTGCGCGCATGCGTATTTGTTAACAAAATGAATATGAATGAATATCATA
ATCCACAAAGCATATAATCCATAGCATATACTCAAGCTCGGTGTCGATGTGTTC
GTCAAACGATATATGGACTTAGCCATCTCGTACAAGGGACTAATTATTTATGCC
TGTTTCTGTGTTTTCGTTTTCCTTAATTTTTTCTCTATTCGCCAACTAAATCAGGA
AAGCCGTTACTACATATCAATCAATTTCGATTATATTCTAAAACTTCTTCTAAAA
AAAAATATTTTGGATATTCAATTATTTTCAAAAAAATTTCTAAAAAATTAAACA
TTTTTTTCTGACGTCGCACGTTTTTTCTAATTTCTTTCTGCGAAACTTTTCAAAA
TCATTTAACAAAATACAAGCCTCTAGGATAGATTCACATAAATTACCAAATCTT
AGATCTCAGGAGACTCATTTGCTTAATTTAATCCTTGTCATCATTTCTAGTATCG
TATTTATTTTTTTTTATCTTAATACATTTATATCATTTAGTGTGACAAATAGAGAG
CATCTAACAGATGAAAAATAATCACATCAATTGGCGCACAAGAGAATTCGCA
GATACTCAAATACTTTCGTTGGAAAAAGATACAAAAAACAGGGTGCAACAAC
TGCACTTAATAGGAAATTACTCACACGAACACAAACTTTTTGCTGCACTTTTG
CTGCATACACGTATTAATAAAATACACTCTCACTGTTTTGCCTTAAGGAAATGT
TCGTATGACACAATACAACCAAGATAATCGCACAGTTTGGCTTTGGCAATCAA
TTTATGCGAAAATAACATGCCCACCAGGAACATAAACACGAACATACTGCCG
AAAAATAATCTAGCACAGAAAACACGTTAACAAGGATAGCAGTAACAACAA
GAATGCAACAACAACAACAACAACAACAACATGTTCATTTCCAGCCCGATCA
TTTCTTACATGACAATTTTTCATTTCCACATTTTGCGAGCCGGTAACAAAACAG
AATCGCTACGCTGCTAATGAAAATAATTCAAAAATAAACTGAGCCAATGCCA
AACCGAAACAAGAACAACAACACGCGTACACACACATAGCCACAGTAACAG
ATTCCCATGCAAATACACTGAGAATATCCTTAAAGCCAGACGAAAAATTTCGA
TTGAAAAATGTTAAGTATTTGTAATCCGGTTTAATTATTCTTAAATCACTTGTGA
AACAAATCAGTTCAATATAATACTAGTAAAATATATTGTAACTTGTAAAATTTTT
CATCTGTAAAAACTGGGACAAATTTACATAAGAGTACGCGTCCAATCCAATTC
AAACTCAGGCGAGCAGCGAGCCAGTTAAAATCAACATGACATGAGTTTACAA
CCAAAAAAAAAAAAAAAAAAAACAGTGAAAAAACGGAAATATACATACAGC

ACAACATACACATGTCTATACATATGTAAATTGTAAATGGTATACTATAACGAG
ATATATAAAAAGAACACAAACAACTCGATAGACCCGTTCACGTTTTGGGTTAA
TTGTGTACCATCTATAAATACCGCCATGCACATAAGTATTCGTATTTAGAGTTG
AGTTTGTGTGTGTGCCAAGAATATACAAGTACGAAAATCCAGGTTGACCCTTT
TAGAACCCTTTTTTGCAATGCCAAAATCCCCACCAACACAAAAACAGAGTTA
TGGAAATTGCATAATCCACACGAAATTTTATTTTCCTTTTCGATTACGCTTGAGT
TTTTGATTCGTTTTTAAGTTCAAATTGTGTTTGAGTTTTTGTTCTGGGCGCGCCA
GAAGCCGAGTGGAAACCAAATCTGCTAGAATAAAGCCAGGAGAAGATTGCA
GGCAACCCACGAGAACCGTGTGCAATTTTCACAGCATAAATAAAACACACAT
AGCCTTTTAGAAAATACGTATACGCCATGTAGACTCGTATCAGCTAGCACCAC
TTAGCGTGCGTTAAAGTACTGGCAAAGTACCCAAAAAGCAAGCAGTGTGCGC
GCCCTATAAATATTTATAACTACTTACAAATGTTATTACCTGCTACTACTACTAC
TACAACAACTATTGTTATTACATTTGCATTTGCATTTACCTTTAACATTTACATTT
ACATGATTTCCTGTTTAATTTTTGGTTCCATGTCGGCCACGTGACGCGCTTGAA
GATAATGATATGCAAGACTGAGAGAAACAACTCGAGTAGTGGTCCTTATCCGC
CCCAAGCCCCCAAAAAAAAAAAAACACTATCCATACCCACACACACACACAG
CCGCCCACCCACAGATACAAAAGCACACAGATACAAACAGAGCAAACACAG
CCAAATTGTTTGTCGTTTTACAATCCAATCCAGTCCCCCTATCGACTCCGATTC
TAAACAATCCAATCGTAGCCAAAAACGCTTAGCTTTGAGGCACAAAAGCAAA
AGCTGAAACAAAAGAGCCAAGCCAAAAAAGCCAACGATATACAAATGTGTA
TACTGTGTATAAAGCGTACAAATAACAATCTGCCAGCCTGTTGCCATAGATGC
CGTAGTAGTCTAGTAATAATAACCCAACCATCCCAGTTTAGTAGCCAGAAAAA
TGCCAAGCTACTTCACAATCACAGCAACGAATCCATACGAATATTTGTGAGAG
GTTTCTGTACTACTTAAATGCTTTACCCGATCCCAAGAACCCTTTCTATTTCTAA
TGTAAATACGCTTAGTAATTTCATTTAATTCTGTTTGTTTTTTAGTTTTATTAAAG
ATCTCAATGCTGATCTATCGAAAAAAAAAAAAAAACAAAACGCTGTATAAAGA
TTCAATTCGATGTTATTGCATGTTGTCTGAAGTCCCGCGACCTTTTGTCCCATCT
TCCTGCATAAATTAATAACTGAAAATTGTTGCACGTTATGAATTCTTCTTGTTTT
CCATTTGACGCAAGTTCTATCCTCGCGTGTTTGTTAAGTGTATCTGTTGTGCCC

AAAATGAATATAACTTTCTGCCTAGTTTTCACAAATTCTCTCTATCGATTTCTCT

ATGAATTTATTTTAGTTGATTGGAATATATATTTTTGAACACACAAGACGTTTAG

TTAGTAGTCTATTAGTTAACTAGTTACGTAAGTTCTCACTGCCAATGTTTACTAA

TATGAATTACCTCTCAATCTCTTTTTGCTACCTTTTCCCCCAATTAACGAAAACC

GTATCGTATCGTGTCGAATCGAATCGAATTGAATCAAATTGTGAATTGAATAGG

ACTTCACATTGGATTTTTACTTTCGTCAATTTTGGACCGATCCTCGTTTAGCGTA

TAGAAAACGACCTGGTGTAGAAACACTATCGGTTGGATCAGAGTTCATTAAG

AATATTTGGGTACCTGACACCTTTTTTGTAAATGAAAAACAATCATATTTTCAC

ATTGCAACAACCAGTAATGAATTCATACGTGTGCATCATTCTGGATCGATAAC

AAGAAGTATTAGGGGCCCGCGGTTCGAAGGTAAGCCTATCCCTAACCCTCTCC

TCGGTCTCGATTCTACGCGTACCGGTCATCATCACCATCACCATTGAGTTTAA

ACCCGCTGATCAGCCTCGACTGTGCCTTCTAAGATCCAGACATGATAAGATA

CATTGATGAGTTTGGACAAACCACAACTAGAATGCAGTGAAAAAAATGCTTT

ATTTGTGAAATTTGTGATGCTATTGCTTTATTTGTAACCATTATAAGCTGCAAT

AAACAAGTTAACAACAACAATTGCATTCATTTTATGTTTCAGGTTCAGGGGG

AGGTGTGGGAGGTTTTTTAAAGCAAGTAAAACCTCTACAAATGTGGTATGGC

TGATTATGATCAGTCGACCTGCAGGCATGCAAGCTTGGCGTAATCATGGTCA

TAGCTGTTTCCTGTGTGAAATTGTTATCCGCTCACAATTCCACACAACATACG

AGCCGGAAGCATAAAGTGTAAAGCCTGGGGTGCCTAATGAGTGAGCTAACT

CACATTAATTGCGTTGCGCTCACTGCCCGCTTTCCAGTCGGGAAACCTGTCGT

GCCAGCTGCATTAATGAATCGGCCAACGCGCGGGGAGAGGCGGTTTGCGTAT

TGGGCGCTCTTCCGCTTCCTCGCTCACTGACTCGCTGCGCTCGGTCGTTCGGCT

GCGGCGAGCGGTATCAGCTCACTCAAAGGCGGTAATACGGTTATCCACAGA

ATCAGGGGATAACGCAGGAAAGAACATGTGAGCAAAAGGCCAGCAAAAGG

CCAGGAACCGTAAAAAGGCCGCGTTGCTGGCGTTTTTCCATAGGCTCCGCCC

CCCTGACGAGCATCACAAAAATCGACGCTCAAGTCAGAGGTGGCGAAACCC

GACAGGACTATAAAGATACCAGGCGTTTCCCCCTGGAAGCTCCCTCGTGCGC

TCTCCTGTTCCGACCCTGCCGCTTACCGGATACCTGTCCGCCTTTCTCCCTTCG

GGAAGCGTGGCGCTTTCTCATAGCTCACGCTGTAGGTATCTCAGTTCGGTGTA

GGTCGTTCGCTCCAAGCTGGGCTGTGTGCACGAACCCCCCGTTCAGCCCGAC
CGCTGCGCCTTATCCGGTAACTATCGTCTTGAGTCCAACCCGGTAAGACACG
ACTTATCGCCACTGGCAGCAGCCACTGGTAACAGGATTAGCAGAGCGAGGT
ATGTAGGCGGTGCTACAGAGTTCTTGAAGTGGTGGCCTAACTACGGCTACAC
TAGAAGAACAGTATTTGGTATCTGCGCTCTGCTGAAGCCAGTTACCTTCGGA
AAAAGAGTTGGTAGCTCTTGATCCGGCAAACAAACCACCGCTGGTAGCGGTG
GTTTTTTTGTTTGCAAGCAGCAGATTACGCGCAGAAAAAAAGGATCTCAAGA
AGATCCTTTGATCTTTTCTACGGGGTCTGACGCTCAGTGGAACGAAAACTCAC
GTTAAGGGATTTTGGTCATGAGATTATCAAAAAGGATCTTCACCTAGATCCTT
TTAAATTAAAAATGAAGTTTTAAATCAATCTAAAGTATATATGAGTAAACTT
GGTCTGACAGTTACCAATGCTTAATCAGTGAGGCACCTATCTCAGCGATCTGT
CTATTTCGTTCATCCATAGTTGCCTGACTCCCCGTCGTGTAGATAACTACGAT
ACGGGAGGGCTTACCATCTGGCCCCAGTGCTGCAATGATACCGCGAGACCCA
CGCTCACCGGCTCCAGATTTATCAGCAATAAACCAGCCAGCCGGAAGGGCC
GAGCGCAGAAGTGGTCCTGCAACTTTATCCGCCTCCATCCAGTCTATTAATTG
TTGCCGGGAAGCTAGAGTAAGTAGTTCGCCAGTTAATAGTTTGCGCAACGTT
GTTGCCATTGCTACAGGCATCGTGGTGTCACGCTCGTCGTTTGGTATGGCTTC
ATTCAGCTCCGGTTCCCAACGATCAAGGCGAGTTACATGATCCCCCATGTTGT
GCAAAAAAGCGGTTAGCTCCTTCGGTCCTCCGATCGTTGTCAGAAGTAAGTT
GGCCGCAGTGTTATCACTCATGGTTATGGCAGCACTGCATAATTCTCTTACTG
TCATGCCATCCGTAAGATGCTTTTCTGTGACTGGTGAGTACTCAACCAAGTCA
TTCTGAGAATAGTGTATGCGGCGACCGAGTTGCTCTTGCCCGGCGTCAATAC
GGGATAATACCGCGCCACATAGCAGAACTTTAAAAGTGCTCATCATTGGAAA
ACGTTCTTCGGGGCGAAAACTCTCAAGGATCTTACCGCTGTTGAGATCCAGTT
CGATGTAACCCACTCGTGCACCCAACTGATCTTCAGCATCTTTTACTTTCACC
AGCGTTTCTGGGTGAGCAAAAACAGGAAGGCAAAATGCCGCAAAAAAGGG
AATAAGGGCGACACGGAAATGTTGAATACTCATACTCTTCCTTTTTCAATATT
ATTGAAGCATTTATCAGGGTTATTGTCTCATGAGCGGATACATATTTGAATGT
ATTTAGAAAAATAAACAAATAGGGGTTCCGCGCACATTTCCCCGAAAAGTGC

CACCTGACGTCTAAGAAACCATTATTATCATGACATTAACCTATAAAAATAG
GCGTATCACGAGGCCCTTTCGT

Supporting Text

Text S2 Amino acid sequences used for homology modelling of CG30122 and Rbp1-like

>CG30122

MDVAKLEKMKVVDLRNELQSRGLDTKGVKAVLVERLRAYVEGGAGDGENAPVT
PSRRQRRTRSMSRSPSPVQAAPVAAEPVLDTLEEEEQPEDKTVPQPEPESEQPAAEP
EPEQSEPEEAEPAAAVTEDTTVNQAVNEESQPEPEEFDEKSETDDKQETIEEAVPAV
VPQNEVADEPMEEDHDAAPEEQEPTQTEEPVEEKPAESTVAEHQSNGDSQKMDV
DEEDSAAPKTAEETEPAAKPEDQPPERRKRSHSRSKSRSRSGSRSSKHRGSVGDKRE
SKAAAEERTVPEDEPTIEENKVGLSWLDSDLHLRIDPTTFASAKPLTSEIYSLIWSGA
RANYGVREGKVCFEVRLSEESVPENSHYFRDEPHVRGFRVGFSMPKSSLLLGEAEH
SFGYCETGRKATQSEFTDYGKPYQLDDVIGCYLDLESEPCTINYTLNGEDLGVAFEF
EKSILGEEGALFPHIVTKGYEYLVNFSDTEQLLVNAERPTRKRRKPRKDEDKDKDD
DKDDNDGEKWKVLDEATADDDEVEKKDEDGKASSEKDEEEAEDPEKAKEDEAA
PKTETEAETKVEAPAEAAKPESEATEASETAETSTEKSSETAAVSNGDAAAEQAND
EKASEDKKPSENNDEEDEDGPSPNKRPKTDGDSEKAESEKEKDRSQTTEDEYEDV
VPEPRDTAALLEGYVLIGLVPVEKLVSGPQRVGSRKECEVILLVGLPGAGKTHWAH
KHVAENADKRYELIGPDAFISKMTIDGASRKTVHKGRWDKVYEICLNSLAALEDI
AMKRRRNFILDQTNVYASAQRRKMKGFNDFKRIAVVCIPSEDELKRRIAEKEEKG
NAFTVKESTINNLRANFTLPSLEFGWFDDINYTELTGDEAKSEVKKYNEKGKKAID
AERSRDKRSRGSRDNYRRDDRNRNRYNDDRRRDYGGQRHESRWSDSRRGGGGG
GYSSNSGGGGSRGYDNRRNYNSGSGGQQNWVQNSRRSGYDDRGYGGGGSGSRG
YDNRNRGYAGGSGGGGGGGGGGGGGGGQQNWMQNNRRSGYDDRGYGSSRDY
RDRDRGNDRSRMGSNDRNRGSSQSSYRSGGGTHQQRDFRPGHRDTKEDSRGGYE
RSAGQSLPKYGNNTAVGGGYDQYKQQGGAPGGGKASLSGKWSTYSQHHQQQQ
APQHQQTGIWQTQQQSQQYQQPQQAAAGQQQYWAYNQMQTGYGNQQAWQS

ADPQQQQQWMSWWQQQQQGSGGAAAGNASGGSGVNVGGGAGNNDGGAA
NHYWSQYSYSTQSNAGADKK

>Rbp1-like
MPRYREWDLACKVYVGNLGSSASKYEIENAFSKYGPLRNVWVARNPPGFAFVEFE
DRRDAEDATRGLDGTRCCGTRIRVEMSSGRSREGRGGGGGGGGGGGGSGGGGR
GGGSGARAGGGGRAGDGGGRYRIKSSSSTTTSTTRTTTTTTSIIIIIMIIKRTTKKKEK
TTTATTTRTSTTPLPSTTTTKRTTTTATTRTTSTTADPAPPITHISPTTTTTTATTTPPPC
NNNLLCGPNQKFNKFDIFR

Supporting Figures

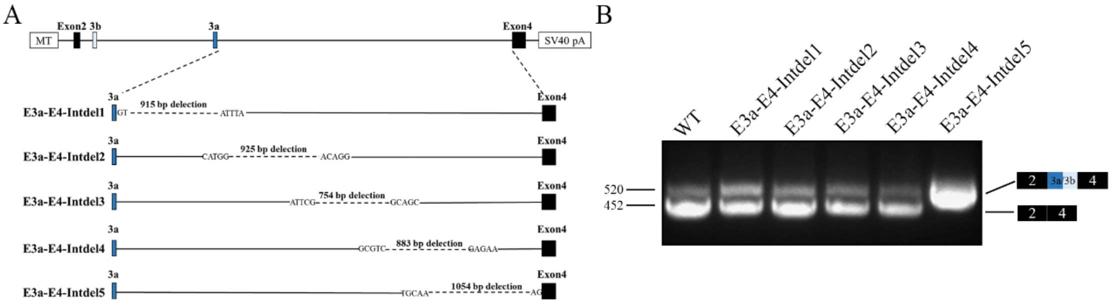

Figure S1. Segment deletion analysis of the intron between E3a and E4 of *DmRdl*. (A) Schematic of the five deletion constructs generated by dividing the intron between E3a and E4 into five segments (segments 1–5), corresponding to E3a-E4-Intdel1 to E3a-E4-Intdel5. Dashed lines indicate deleted regions, and their lengths (bp) are indicated. (B) Splicing patterns of the WT and deletion constructs in S2 cells, as detected by RT-PCR using primers *DmRdl*-det-F and *DmRdl*-det-R.

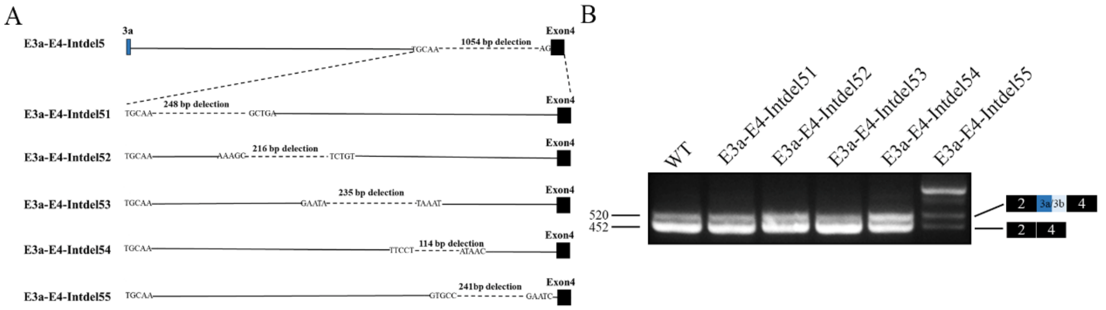

Figure S2. Fine-mapping analysis of segment 5 for MEAS of *DmRdl* E3. (A) Schematic of the five deletion constructs generated by dividing segment 5 into five subsegments (segments 51–55), corresponding to E3a-E4-Intdel51 to E3a-E4-Intdel55. Dashed lines indicate deleted regions, and their lengths (bp) are indicated. (B) Splicing patterns of the WT and deletion constructs in S2 cells, as detected by RT-PCR using primers *DmRdl*-det-F and *DmRdl*-det-R.

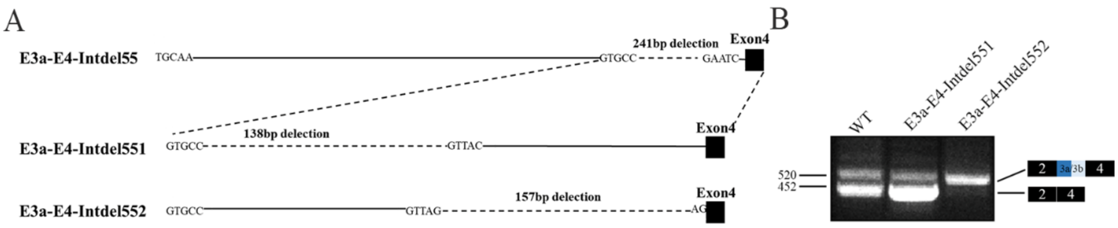

Figure S3. Fine-mapping analysis of the segment 55 and its role in MEAS of *DmRdl* E3. (A) Schematic of the five deletion constructs generated by dividing segment 55 into two smaller segments (segments 551–552), corresponding to E3a-E4-Intdel551 to E3a-E4-Intdel552. Dashed lines indicate deleted regions, and their lengths (bp) are indicated. (B) Splicing patterns of the WT and deletion constructs in S2 cells, as detected by RT-PCR using primers *DmRdl*-det-F and *DmRdl*-det-R.

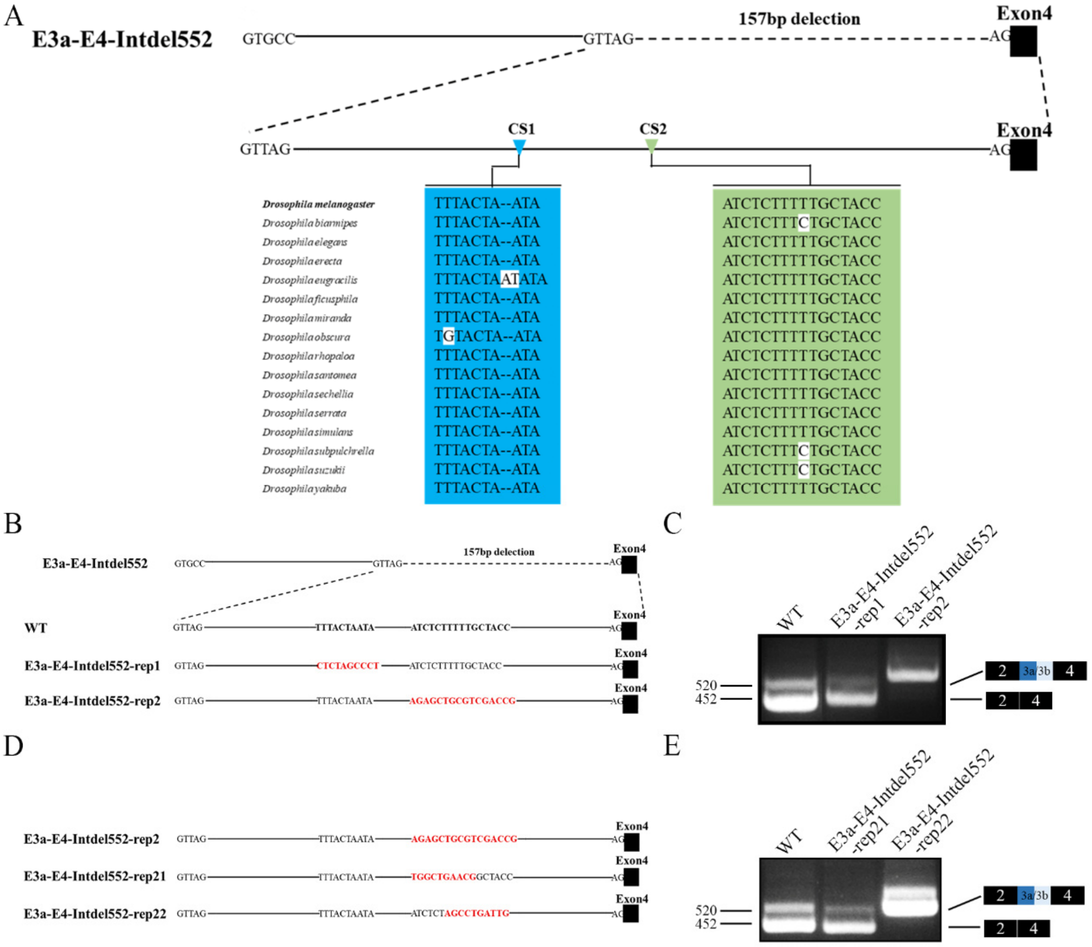

Figure S4. Identification of conserved intronic elements within segment 552 and replacement analysis of their roles in the MEAS of *DmRdl* E3. (A) Conserved intronic sequences CS1 and CS2 identified within the E3a-E4-Intdel552 deletion region. (B) Schematic of replacement constructs generated by individually replacing CS1 or CS2 within segment 552, corresponding to E3a-E4-Intdel552-rep1 and E3a-E4-Intdel552-rep2. The replaced regions are indicated in red. (C) Splicing patterns of the WT and replacement constructs shown in (B) in S2 cells, as detected by RT-PCR using primers *DmRdl*-det-F and *DmRdl*-det-R. (D) Schematic of replacement constructs generated by further subdividing CS2, corresponding to E3a-E4-Intdel552-rep21 and E3a-E4-Intdel552-rep22. The replaced regions are indicated in red. (E) Splicing patterns of the WT and replacement constructs shown in (D) in S2 cells, as detected by RT-PCR using primers *DmRdl*-det-F and *DmRdl*-det-R.

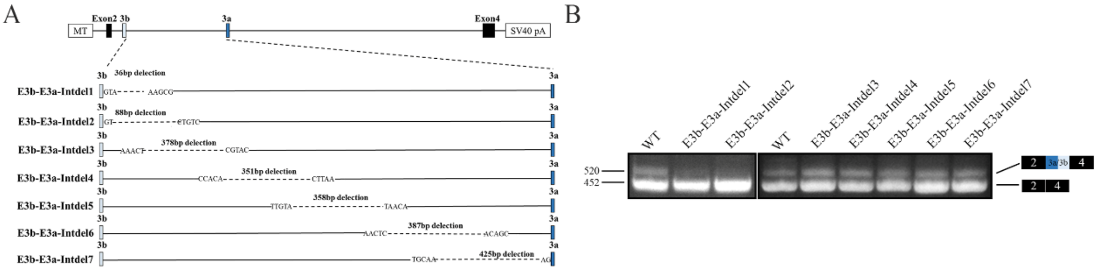

Figure. S5. Segment deletion analysis of the intron between E3b and E3a of *DmRdl*. (A) Schematic of the seven deletion constructs generated by dividing the intron between E3b and E3a into seven segments (segments 1–7), corresponding to E3b-E3a-Intdel1 to E3b-E3a-Intdel7. Dashed lines indicate deleted regions, and their lengths (bp) are indicated. (B) Splicing patterns of the WT and deletion constructs in S2 cells, as detected by RT-PCR using primers *DmRdl*-det-F and *DmRdl*-det-R.

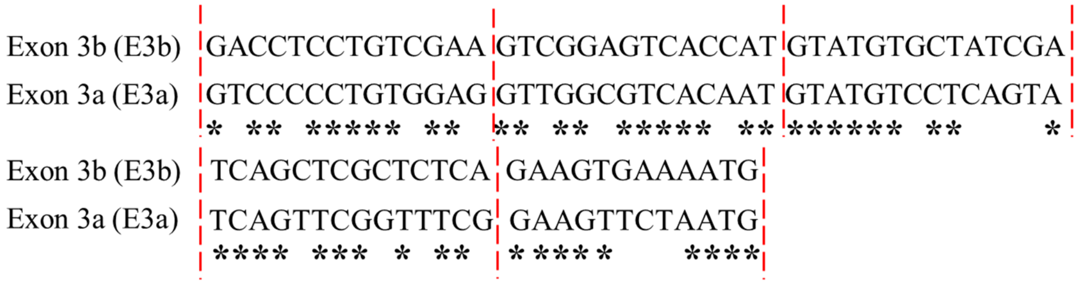

Figure S6. Sequence alignment of *DmRdl* E3b and E3a.

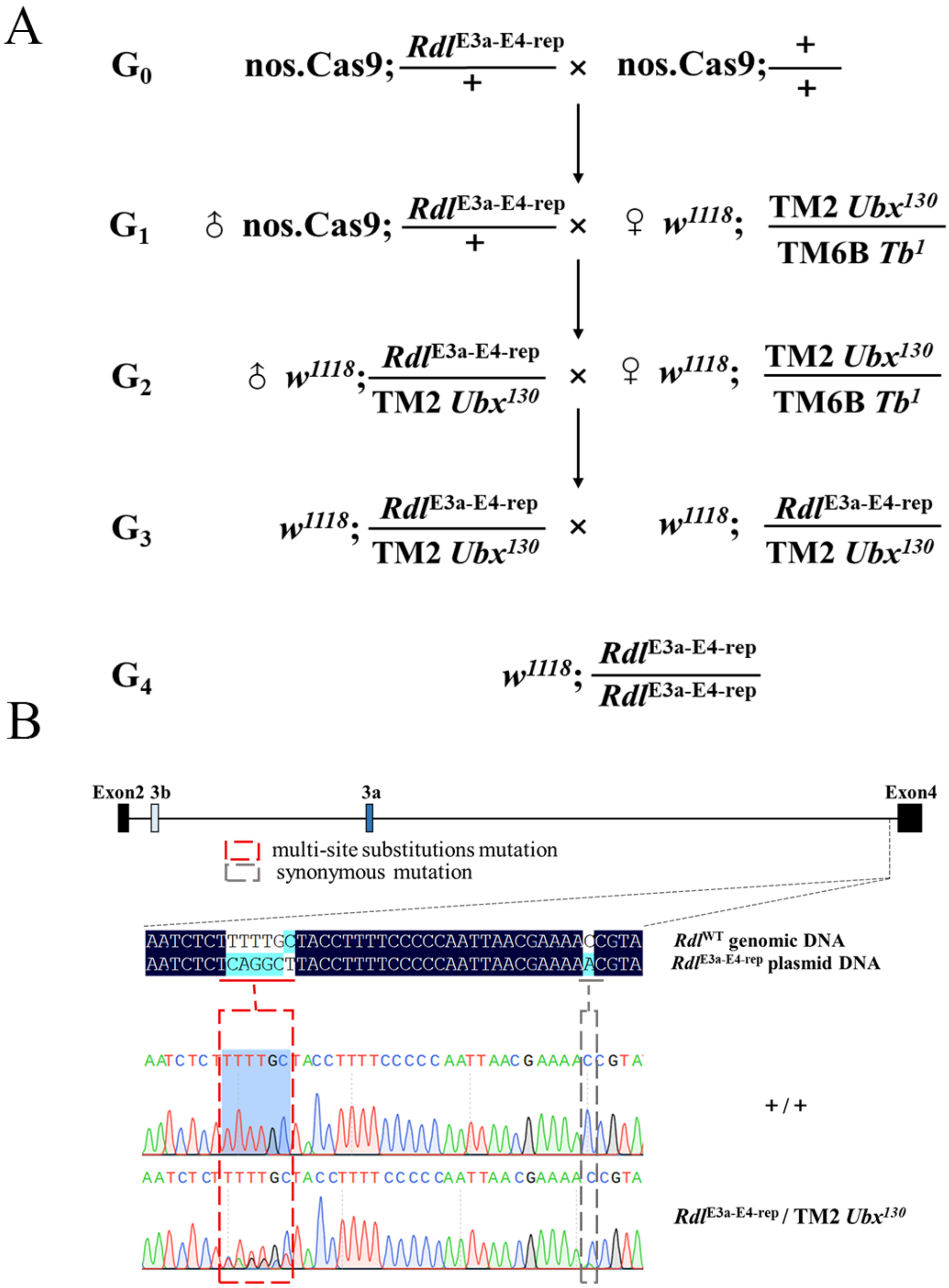

Figure S7. Generation of *Rdl*$^{E3a\text{-}E4\text{-}rep}$ by CRISPR/Cas9 genome editing. (A) Crossing procedures for establishing homozygous *Rdl*$^{E3a\text{-}E4\text{-}rep}$ flies. (B) Heterozygous *Rdl*$^{E3a\text{-}E4\text{-}rep}$ flies were confirmed by genomic DNA sequencing. Gray box indicates synonymous mutations designed to prevent repeated editing; the red box indicates the replacement in the IRE1.

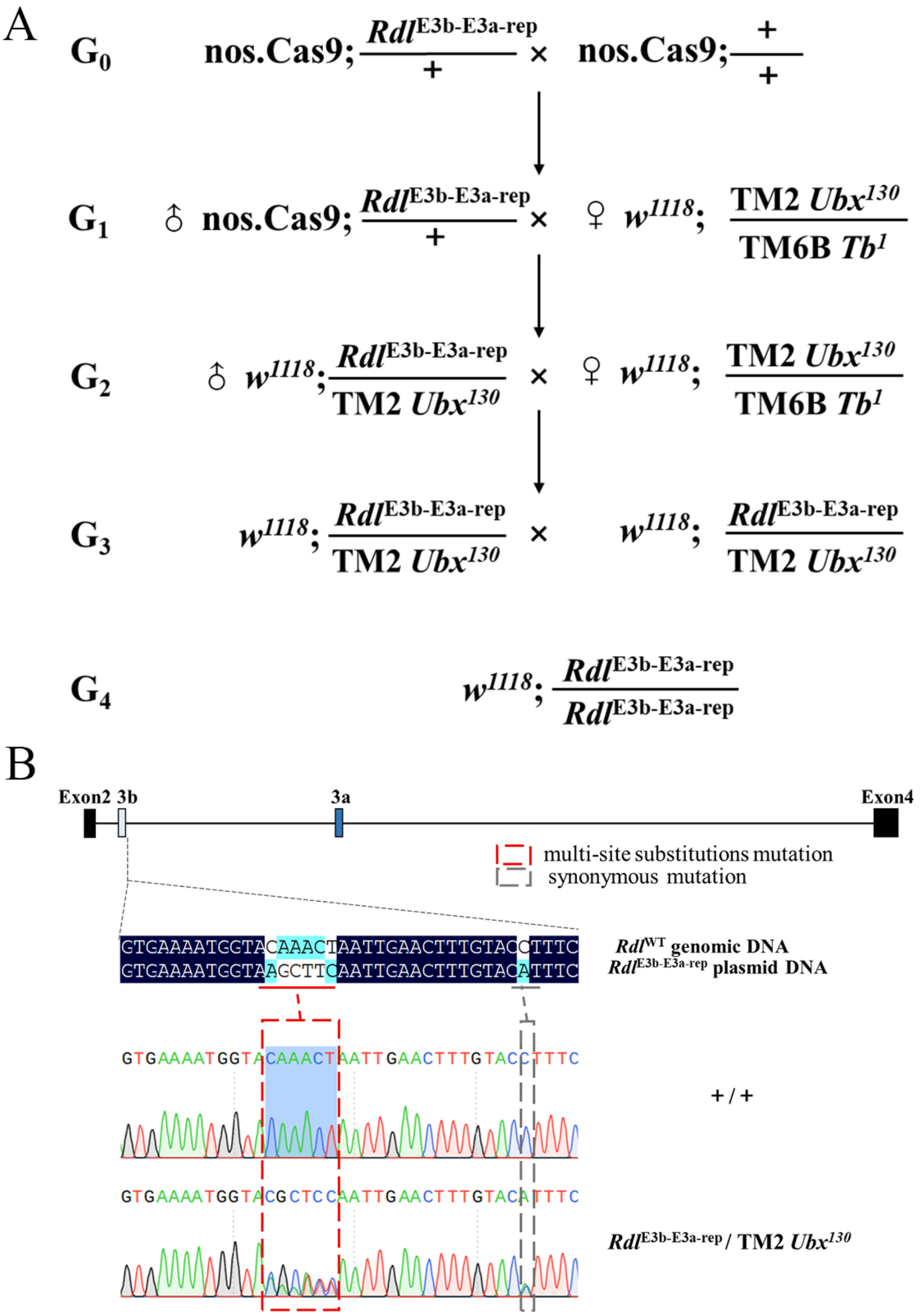

Figure S8. Generation of *Rdl*$^{E3b\text{-}E3a\text{-}rep}$ by CRISPR/Cas9 genome editing. (A) Crossing procedures for establishing homozygous *Rdl*$^{E3b\text{-}E3a\text{-}rep}$ flies. (B) Heterozygous *Rdl*$^{E3b\text{-}E3a\text{-}rep}$ flies were confirmed by genomic DNA sequencing. Gray box indicates synonymous mutations designed to prevent repeated editing; the red box indicates the replacement in the IRE2.

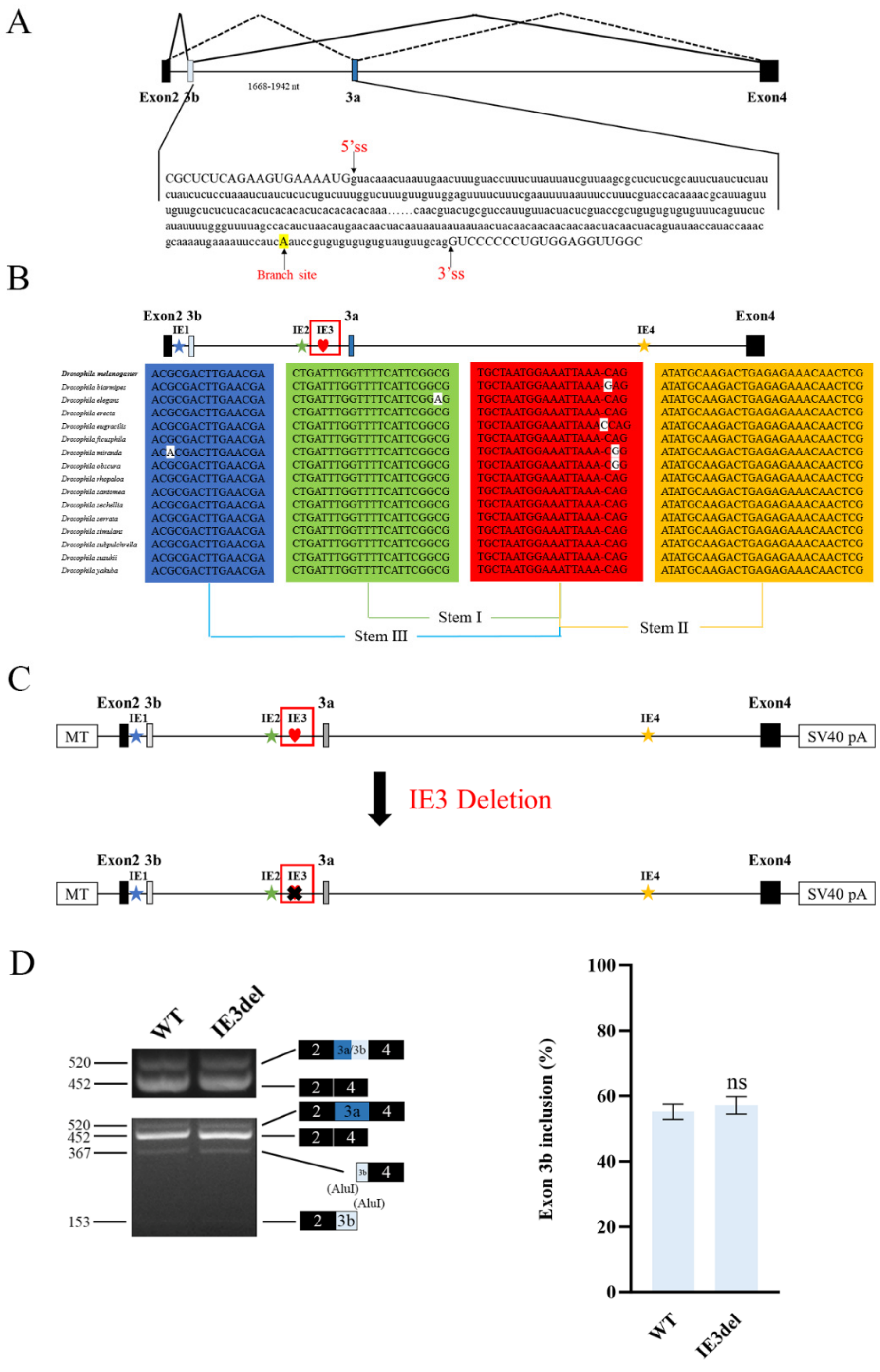

Figure S9. Steric hindrance and competing RNA secondary structure models do not account for the MEAS of *DmRdl* E3. (A) Schematic of the *DmRdl* E2-E4 pre-mRNA region. E3b, E3a and introns are represented by the light blue box, the dark blue box, and lines, respectively. (B) Conserved intronic elements and predicted RNA secondary structure Stems I -III within the *DmRdl* E3 cluster. (C) Schematic of the IE3 deletion in *DmRdl*. (D) Splicing patterns of the WT and IE3del construct transfected into S2 cells were detected by RT-PCR. PCR products were digested with Alu I and separated by 3% agarose gel. ns indicates no significant difference ($P > 0.05$).

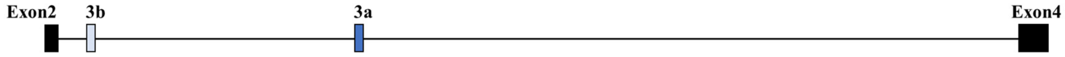

Figure S10. Schematic of the *DmRdl* pre-mRNA region spanning E2 to E4.

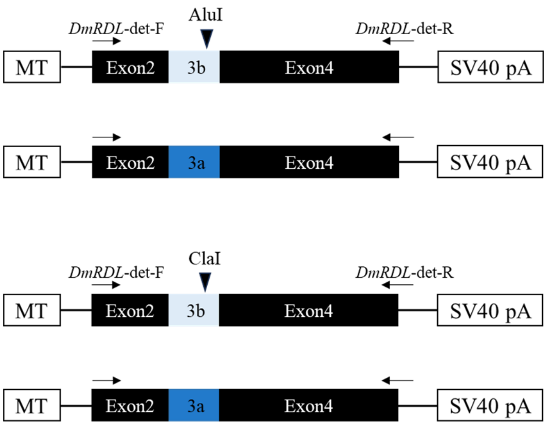

Figure S11. Scheme for assaying E3b/E3a inclusion ratio.
